# Bat and Lyssavirus Exposure among Humans in Area that Celebrates Bat Festival, Nigeria, 2010 and 2013

**DOI:** 10.3201/eid2607.191016

**Published:** 2020-07

**Authors:** Neil M. Vora, Modupe O.V. Osinubi, Lora Davis, Mohammed Abdurrahman, Elizabeth B. Adedire, Henry Akpan, Abimbola F. Aman-Oloniyo, Solomon W. Audu, Dianna Blau, Raymond S. Dankoli, Ajoke M. Ehimiyein, James A. Ellison, Yemi H. Gbadegesin, Lauren Greenberg, Dana Haberling, Christina Hutson, Jibrin M. Idris, Grace S.N. Kia, Maruf Lawal, Samson Y. Matthias, Philip P. Mshelbwala, Michael Niezgoda, Albert B. Ogunkoya, Abiodun O. Ogunniyi, Gloria C. Okara, Babasola O. Olugasa, Okechukwu P. Ossai, Akin Oyemakinde, Marissa K. Person, Charles E. Rupprecht, Olugbon A. Saliman, Munir Sani, Olufunmilayo A. Sanni-Adeniyi, P.S. Satheshkumar, Todd G. Smith, Mariat O. Soleye, Ryan M. Wallace, Sebastian K. Yennan, Sergio Recuenco

**Affiliations:** Centers for Disease Control and Prevention, Atlanta, Georgia, USA (N.M. Vora, M.O.V. Osinubi, L. Davis, D. Blau, J.A. Ellison, L. Greenberg, D. Haberling, C. Hutson, M. Niezgoda, M.K. Person, C.E. Rupprecht, P.S. Satheshkumar, T.G. Smith, R.M. Wallace, S. Recuenco);; Ahmadu Bello University, Zaria, Nigeria (M. Abdurrahman, S.W. Audu, A.M. Ehimiyein, G.S.N. Kia, M. Lawal, A.B. Ogunkoya, M. Sani);; African Field Epidemiology Network, Abuja, Nigeria (E.B. Adedire, J.M. Idris, G.C. Okara);; Federal Ministry of Health, Abuja (H. Akpan, A. Oyemakinde, O.A. Sanni-Adeniyi);; Walden University, Abuja (A.F. Aman-Oloniyo); World Health Organization, Borno, Nigeria (R.S. Dankoli);; Nigerian Institute of Science Laboratory Technology, Ibadan, Nigeria (Y.H. Gbadegesin);; Ministry of Health, Kaduna State, Kaduna, Nigeria (S.Y. Matthias);; University of Queensland, Brisbane, Queensland, Australia (P.P. Mshelbwala);; University of Ibadan, Ibadan (A.B. Ogunkoya, B.O. Olugasa);; Nigeria Centre for Disease Control, Abuja (A.O. Ogunniyi, S.K. Yennan);; Ministry of Health, Enugu State, Enugu, Nigeria (O.P. Ossai);; Ministry of Agriculture and Natural Resources, Ilorin, Nigeria (O.A. Saliman);; Federal Ministry of Agriculture and Rural Development, Abuja (M.O. Soleye)

**Keywords:** Lyssavirus, viruses, bats, Nigeria, zoonotic disease, rabies, zoonoses

## Abstract

Using questionnaires and serologic testing, we evaluated bat and lyssavirus exposure among persons in an area of Nigeria that celebrates a bat festival. Bats from festival caves underwent serologic testing for phylogroup II lyssaviruses (Lagos bat virus, Shimoni bat virus, Mokola virus). The enrolled households consisted of 2,112 persons, among whom 213 (10%) were reported to have ever had bat contact (having touched a bat, having been bitten by a bat, or having been scratched by a bat) and 52 (2%) to have ever been bitten by a bat. Of 203 participants with bat contact, 3 (1%) had received rabies vaccination. No participant had neutralizing antibodies to phylogroup II lyssaviruses, but >50% of bats had neutralizing antibodies to these lyssaviruses. Even though we found no evidence of phylogroup II lyssavirus exposure among humans, persons interacting with bats in the area could benefit from practicing bat-related health precautions.

Bats are vital to many ecosystems and provide benefits to humans (*1*). However, under certain circumstances, bats may pose a risk to human health, as they host several zoonotic pathogens (*2*). Humans should therefore avoid bat contact unless appropriate precautions are taken. Among the most concerning batborne pathogens are viruses within the genus *Lyssavirus*. Previously unimmunized humans exposed to any of the >16 currently recognized and putative lyssaviruses (typically through a bite from an infected animal) will have 1 of 3 outcomes. First is a complete lack of any lyssavirus infection, characterized by the absence of both illness and lyssavirus-neutralizing antibody production. Second is a productive lyssavirus infection, characterized by a fatal encephalitis known as rabies (*3*). A human with rabies may produce lyssavirus-neutralizing antibodies in the end stages of illness as the disease progresses, although this response is typically inadequate for viral clearance (*4*). Third is an abortive lyssavirus infection (sometimes termed an exposure) characterized by the absence of frank encephalitis but with production of lyssavirus-neutralizing antibodies. Although rarely documented, the prevalence of abortive lyssavirus infections among some Amazonian communities whose members experience frequent bites from vampire bats has challenged the paradigm that lyssavirus infections are nearly always productive and therefore fatal (*5*).

The various lyssaviruses sort into different phylogroups (*6*). Phylogroup I includes rabies virus, Duvenhage virus, and several others. Rabies can be prevented after exposure to phylogroup I lyssaviruses with prompt administration of postexposure prophylaxis (PEP) that includes wound cleansing, rabies vaccine, and, when indicated, rabies immune globulin (*3*,*7*,*8*). Phylogroup II includes Lagos bat virus, Shimoni bat virus, and Mokola virus. These viruses are phylogenetically and antigenically distant from phylogroup I members (*9*). West Caucasian bat virus and Ikoma lyssavirus are even more distant lyssaviruses (*10*,*11*). The rabies vaccines available for use in the previously described PEP regimen may not be effective against non–phylogroup I lyssaviruses (*10*–*12*). Evidence of abortive lyssavirus infections outside the Amazon is limited, but they could possibly occur wherever humans frequently interact with infected animals (*5*,*13*,*14*). 

Twice a year in the Idanre area of Nigeria, a 1-day bat festival takes place in which boys and men enter into designated caves to capture bats, typically with their bare hands (*15*) (Figure 1). Captured bats are cooked and eaten, sold in markets, and used in cultural ceremonies. Pathogen spillover from bats to humans might occur during these festivities, given that some Nigerian bats harbor lyssaviruses such as Lagos bat virus and other pathogens such as *Bartonella rousetti* (*16*–*20*). Furthermore, the most frequently identified bat species roosting in the festival caves is the Egyptian fruit bat (*Rousettus aegyptiacus*), which is a reservoir for Marburg virus and Sosuga virus (*15*,*21*–*23*).

**Figure 1:**
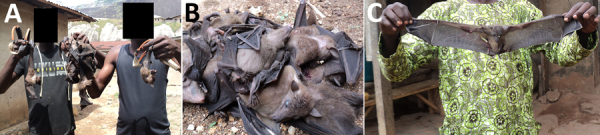
Bat hunters and bats captured during a bat festival, Idanre area, Nigeria, 2013. A) Bat hunters with slingshots and bats captured during a bat festival. B) Bats captured during a bat festival. C) Bat hunter with a bat captured during a bat festival.

We evaluated bat and lyssavirus exposure among humans in the area around Idanre, Nigeria. Our objectives were to determine the prevalence of bat contact, to identify factors associated with bat contact, to assess knowledge about batborne infections and health precautions related to bats, to determine whether febrile illnesses occur following the bat festival, to determine whether abortive lyssavirus infections occur, and to identify whether lyssaviruses circulate among bats in the festival caves.

## Methods

### Study Design

Work with human participants was approved by the Centers for Disease Control and Prevention (CDC), Ahmadu Bello University, and the National Health Research Ethics Committee of Nigeria. All animal sampling was conducted in compliance with a protocol approved by the CDC Animal Institutional Care and Use Committee.

**Figure 2:**
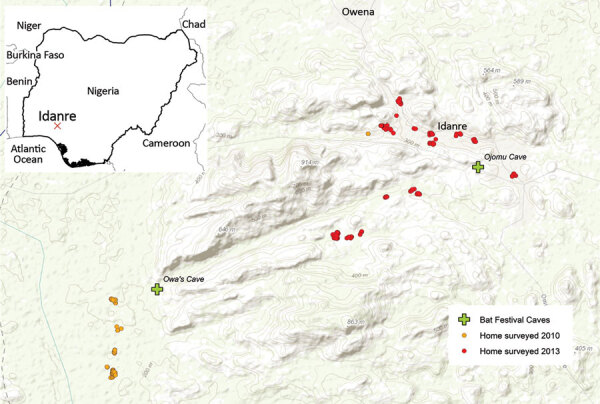
Locations of festival caves and households enrolled in 2 community surveys and a bat hunter survey of bat exposures, Idanre area, Nigeria, 2010 and 2013. Inset map shows location of Idanre area within Nigeria.

Persons eligible to participate were those residing in communities located near the 2 festival caves in the Idanre area (Figure 2). We recruited study participants through community surveys and through a convenience sample; some respondents participated in a follow-up survey. Before enrolling, adults (persons >18 years of age) and mature minors (persons 13–17 years of age who were married, had children, or provided for their own livelihood) provided consent. Persons <18 years of age who were not mature minors had to get guardian consent and provide assent if >7 years of age. We administered study questionnaires verbally and recorded responses electronically. After administering the study questionnaire, we collected blood specimens from participants who agreed.

**Figure 3:**
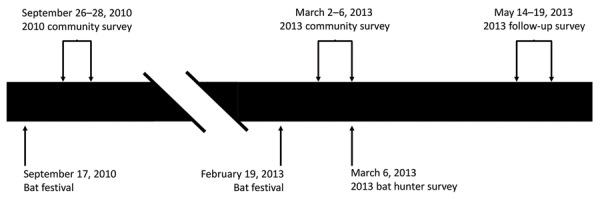
Timeline of events for 2 community surveys, a bat hunter survey, and a follow-up survey of bat exposures, Idanre area, Nigeria, 2010 and 2013.

We completed community surveys during September 26–28, 2010 (2010 community survey; 9–11 days after the September 17, 2010, bat festival) and March 2–March 6, 2013 (2013 community survey; 11–15 days after the February 19, 2013, bat festival) (Figure 3). We enrolled households into the survey from 9 rural villages near the festival caves and from the town of Idanre. Generally, all households within rural villages were offered enrollment in the study. In contrast, Idanre was divided into ≈100 zones, and households from 10 randomly selected zones were offered enrollment in the study. At the time of the household visit, an adult or mature minor had to be present. If consent was provided, this adult or mature minor was considered the main household respondent and was the first person of the household to whom the study questionnaire was administered (Appendix1). We then administered a similar study questionnaire to additional household respondents, who were other consenting or assenting household members. However, to enroll as an additional household respondent, the household member had to be immediately available and either had previously had bat contact (defined as having touched a bat, having been bitten by a bat, or having been scratched by a bat) or had eaten a bat. This requirement was different than that for main household respondents, for whom having had bat contact or having eaten a bat were not requirements for enrollment.

We recruited additional participants outside the community surveys on March 6, 2013 (2013 bat hunter survey) using a convenience sample of bat hunters composed exclusively of persons who actively trapped bats during the bat festival (they may also have trapped bats at other times of the year) (Figure 3). These participants answered the same study questionnaire as main household respondents from the community survey (Appendix 1). Study participants in the community surveys may also have hunted bats (in that they actively trapped bats during and outside the bat festival), but data for these participants were analyzed with other community survey data and handled separately from the 2013 bat hunter survey. Persons who participated in the 2013 community survey or 2013 bat hunter survey and who agreed underwent a follow-up survey during May 14–19, 2013 (2013 follow-up survey; 85–90 days after the February 19, 2013, bat festival took place) (Appendix 1).

### Human Serologic Testing

We stored blood specimens on ice and centrifuged them within 12 hours of collection. We stored serum specimens at −80°C except while in the field and during shipment to the United States, when they were stored on dry ice. We tested serum specimens for neutralizing antibodies against rabies virus, Duvenhage virus, Lagos bat virus, Shimoni bat virus, Mokola virus, and West Caucasian bat virus using a modification of the rapid fluorescent focus inhibition test (*5*,*24*–*26*). We considered serum samples that exhibited complete neutralization of challenge lyssavirus at a 1:5 serum dilution to have detectable neutralizing antibodies to that lyssavirus (*3*).

### Bat Capture, Species Identification, Specimen Collection, and Testing

We captured bats from the 2 festival caves (Figure 2) using nets. Taxonomic identification of bat species was based on morphology. We anesthetized bats by intramuscular injection of ketamine and then euthanized them via cardiac exsanguination. We centrifuged blood specimens within 4 hours of collection. We also collected bat brains. We stored serum and brain specimens at −80°C except while in the field and during shipment to the United States, when they were stored on dry ice.

We tested serum samples for neutralizing antibodies against Duvenhage virus, Lagos bat virus, Shimoni bat virus, Mokola virus, and Ikoma lyssavirus using a microneutralization test (*27*). We considered serum samples that exhibited >50% neutralization of challenge lyssavirus at 1:10 serum dilution to have detectable neutralizing antibodies to that lyssavirus. We tested brains for lyssavirus antigens with the direct fluorescent antibody test using a FITC-labeled monoclonal antibody kit (Fujirebio Diagnostics, https://www.fujirebio.com) (*28*) (Appendix 2).

### Data Analysis

We analyzed data using SAS software (https://www.sas.com) (details in Appendix 2). A p value <0.05 was considered statistically significant.

## Results

Through the community surveys in 2010 and 2013, we enrolled 264 households (254 unique households and 10 that participated in both years) (Table 1). Each enrolled household had a main household respondent; 87 persons from enrolled households participated as additional household respondents. Most of the 2013 respondents also participated in the 2013 follow-up survey (172/217 [79%] from the 2013 community survey and 18/21 [86%] from the 2013 bat hunter survey).

**Table 1:** Characteristics of households enrolled in 2 community surveys of bat exposures, Idanre area, Nigeria, 2010 and 2013

Characteristic	2010 community survey, no. (%)	2013 community survey, no. (%)	Total, no. (%)
Households visited	90	183	273
Households enrolled	90 (100)	174 (95)	264 (97)
Total participants enrolled	134	217	351
Main household respondents*	90 (67)	174 (80)	264 (75)
Additional household respondents*	44 (33)	43 (20)	87 (25)
Mean participants enrolled per household (SD)	1.5 (0.9)	1.2 (0.6)	1.3 (0.7)
Main household respondents*	1.0 (0)	1.0 (0)	1.0 (0)
Additional household respondents*	0.5 (0.9)	0.2 (0.6)	0.3 (0.7)
Mean persons per household (SD)	7.6 (4.7)	8.2 (5.7)	8.0 (5.4)
Persons living within enrolled households	688	1,424	2,112
Male	372 (54)	734 (52)	1,106 (52)
Female	316 (46)	690 (48)	1,006 (48)
Age distribution of persons represented among enrolled households	n = 688	n = 1,424	n = 2,112
<6 y	115 (17)	278 (20)	393 (19)
6‒17 y	162 (24)	419 (29)	581 (28)
>18 y	411 (60)	727 (51)	1,138 (54)
Main material used to build house	n = 90	n = 174	n = 264
Adobe/mud	56 (62)	82 (47)	138 (52)
Cement/brick	33 (37)	92 (53)	125 (47)
Wood	1 (1)	0	1 (0.4)
Openings in house that could allow bats to enter	56 (62)	106 (61)	162 (61)
Households with animals (pets or livestock) (%)	52 (58)	90 (52)	142 (54)
Households with ≥1 animal (pet or livestock) that had been vaccinated against rabies	0 (0)	7 (8)	7 (5)
Households with >1 member who had ever participated in bat festival†	22 (24)	50 (29)	72 (27)
Households with >1 member who had ever had bat contact‡	51 (57)	117 (67)	168 (64)
Households with >1 member who had ever touched a bat	50 (56)	116 (67)	166 (63)
Households with >1 member who had ever been bitten by a bat	14 (16)	30 (17)	44 (17)
Households with >1 member who had ever been scratched by a bat	19 (21)	37 (21)	56 (21)
Households with >1 member who had ever eaten a bat	64 (71)	124 (71)	188 (71)

*Main household respondents are adults or mature minors (persons aged 13–17 y who were married, had children, or provided for their own livelihood) present at the time of household visit who provided consent to participate in the survey; the main household respondent was the first person of the household to whom the study questionnaire was administered. Additional household respondents are other consenting or assenting household members who were immediately available to answer the study questionnaire and either had previously had bat contact or had previously eaten a bat. †This may be an underestimate, as only main and additional household respondents were asked if they had participated in the bat festival. We did not ask if other members of the household had ever participated in the bat festival. ‡Bat contact was defined as having touched a bat, having been bitten by a bat, or having been scratched by a bat.

More than one quarter of enrolled households (72/264; 27%) had ≥1 household member who had ever participated in the bat festival (Table 1). Almost two thirds of enrolled households (168; 64%) had >1 household member who had ever had bat contact. Nearly two thirds of enrolled households (166; 63%) had >1 household member who had ever touched a bat. About one fifth of households had >1 household member who had ever been bitten (44; 17%) or scratched (56; 21%) by a bat. Nearly three quarters of households had >1 household member who had ever eaten a bat (188; 71%).

The enrolled households were composed of 2,112 persons, among whom 213 (10%) were reported to have ever had bat contact, 211 (10%) to have ever touched a bat, 52 (2%) to have ever been bitten by a bat, 66 (3%) to have ever been scratched by a bat, and 265 (13%) to have ever eaten a bat (Table 2). Of 254 main household respondents, 141 (56%) reported having ever had bat contact (Table 3). Factors significantly associated with bat contact included being male (OR 2.08, 95% CI 1.24–3.49), having ever participated in the bat festival (OR 20.17, 95% CI 6.09–66.82), having ever entered a bat cave or bat refuge (OR 31.45, 95% CI 7.45–132.73), having ever prepared a bat as food (OR 9.85, 95% CI 5.37–18.07), and having ever eaten a bat (OR 8.56, 95% CI 4.57–16.03).

**Table 2:** Types of bat exposure among persons living within households enrolled in 2 community surveys of bat exposures, Idanre area, Nigeria, 2010 and 2013

Type of bat exposure	No. (%), n = 2,112
Ever had bat contact*	213 (10)
Ever touched a bat	211
Ever bitten by a bat	52
Ever scratched by a bat	66
Ever eaten a bat	265 (13)

*Bat contact was defined as having touched a bat, having been bitten by a bat, or having been scratched by a bat.

**Table 3:** Factors associated with having ever had bat contact among main household respondents† in 2 community surveys of bat exposures, Idanre area, Nigeria, 2010 and 2013*

Characteristic	Contact, no. (%),‡ n = 141	No contact, no. (%),‡ n = 113	p value	OR (95% CI)
Demographics				
Location				
Mean age (SD)	47 (17)	40 (16)	0.001	–
Age range, y	18–89	17–87	–	–
Median age (interquartile range)	45 (33–60)	35 (28–50)	0.001	–
Age <25 y	13 (9)	19 (17)	0.07	0.50 (0.24–1.07)
Male	100 (71)	61 (54)	0.01	2.08 (1.24–3.49)
Education				
Some secondary or above	67 (48)	42 (37)	0.10	1.53 (0.92–2.54)
Completed secondary or above	41 (29)	30 (27)	0.66	1.13 (0.65–1.97)
Household characteristics				
No. years living in house				
<1 y	15 (11)	16 (16)	0.26	0.65 (0.30–1.38)
<5 y	41 (30)	46 (46)	0.01	0.51 (0.30–0.86)
<10 y	59 (43)	62 (61)	0.005	0.47 (0.28–0.79)
Persons in household				
<5 persons	36 (26)	27 (24)	0.76	1.09 (0.61–1.94)
<10 persons	93 (66)	84 (74)	0.15	0.67 (0.39–1.16)
Main material used to build house				
Adobe/mud	66 (47)	69 (61)	0.04	0.57 (0.33–0.97)
Cement/brick	74 (52)	44 (39)	Ref	Referent
Wood	1 (1)	0 (0)	1.00	0.59 (0.03–inf)
Openings in house that could allow bats to enter	82 (58)	73 (65)	0.30	0.76 (0.46–1.27)
Household with animals (pets or livestock)	84 (60)	55 (49)	0.08	1.55 (0.94–2.56)
Household with >1 animal (pest or livestock) that has been vaccinated against rabies	6 (7)	1 (2)	0.19	4.15 (0.49–35.47)
Other bat-related activities				
Participate in bat festival				
Ever participated	50 (36)	3 (3)	<0.0001	20.17 (6.09–66.82)
First time participated >20 y ago	22 (55)	1 (33)	0.48	2.44 (0.20–29.19)
Participate 2 times/y	22 (45)	1 (33)	0.70	1.63 (0.14–19.18)
Enter a bat cave or bat refuge				
Ever entered	51 (36)	2 (2)	<0.0001	31.45 (7.45–132.73)
Last time entered ≤6 mo ago	16 (31)	1 (50)	0.59	0.46 (0.03–7.78)
Enter >2 times/y	14 (27)	1 (50)	0.50	0.38 (0.02–6.47)
Prepare a bat as food				
Ever prepared	121 (86)	43 (38)	<0.0001	9.85 (5.37–18.07)
Last time prepared ≤6 mo ago	66 (55)	19 (44)	0.21	1.57 (0.78–3.17)
Prepare >2 times/y	28 (23)	4 (9)	0.06	2.94 (0.97–8.93)
Eat a bat				
Ever eaten	124 (88)	52 (46)	<0.0001	8.56 (4.57–16.03)
Last time eaten <1 mo ago	48 (39)	12 (23)	0.049	2.11 (1.01–4.41)
Eat >2 times/y	34 (27)	10 (19)	0.26	1.59 (0.72–3.51)
Knowledge				
Indicated animal bites as mechanism of rabies transmission	87 (62)	54 (48)	0.03	1.73 (1.05–2.86)
Described rabies as severe	94 (67)	55 (49)	0.004	2.11 (1.27–3.51)
Identified bats as a rabies source	4 (3)	2 (2)	0.58	1.62 (0.29–9.01)
Identified dogs as a rabies source	94 (67)	62 (55)	0.06	1.65 (0.99–2.74)
If bitten or scratched by a bat				
Wash wound with soap and water	11 (8)	2 (2)	0.048	4.69 (1.02–21.61)
Seek medical care	38 (27)	35 (31)	0.48	0.82 (0.47–1.42)
Seek a traditional healer or pray	5 (4)	5 (4)	0.72	0.79 (0.22–2.81)
Do nothing	69 (49)	50 (45)	0.46	1.21 (0.73–1.98)
If bitten by a potentially rabid animal				
Wash wound with soap and water	3 (2)	1 (1)	0.44	2.43 (0.25–23.73)
Seek medical care	93 (66)	69 (61)	0.42	1.24 (0.74–2.07)
Seek a traditional healer or pray	7 (5)	3 (3)	0.35	1.91 (0.48–7.58)
Do nothing	22 (16)	18 (16)	0.94	0.98 (0.49–1.92)
History of rabies vaccination	1 (1)	1 (1)	0.87	0.80 (0.05–12.91)
Aware that bats can cause disease other than rabies	8 (6)	4 (4)	0.44	1.62 (0.48–5.54)
Know of reports of illness as a result of bats or being in bat cave	3 (2)	1 (1)	0.45	2.41 (0.25–23.52)

*Bat contact was defined as having touched a bat, having been bitten by a bat, or having been scratched by a bat. OR, odds ratio; –, not applicable or not calculated. †Main household respondents are adults or mature minors (persons aged 13–17 y who were married, had children, or provided for their own livelihood) present at the time of household visit who provided consent to participate in the survey; the main household respondent was the first person of the household to whom the study questionnaire was administered. Additional household respondents are other consenting or assenting household members who were immediately available to answer the study questionnaire and either had previously had bat contact or had previously eaten a bat. ‡Ten of the 264 main household respondents participated in both the 2010 community survey and the 2013 community survey. They were deleted from the 2013 community survey data..

Although more than half of participants with bat contact in the 2010 community survey, 2013 community survey, and 2013 bat hunter survey knew that animal bites are a mechanism of rabies virus transmission or that rabies is severe, they more often attributed dogs as being a rabies source (≥60%) than bats (≤3%) (Appendix 2 Table 1). About 50% of participants with bat contact in the 2010 and 2013 community surveys and 86% of participants in the 2013 bat hunter survey stated that they would do nothing if bitten or scratched by a bat. Among participants with bat contact in the 2010 community survey, 2013 community survey, and 2013 bat hunter survey, only 1%, 2%, and 5%, respectively, had ever received rabies vaccination. Furthermore, only 3%, 7%, and 5% of these participants, respectively, were aware that bats can cause diseases other than rabies.

More main household respondents with bat contact knew that animal bites are a mechanism of rabies virus transmission and that rabies is severe compared with those without bat contact (Table 3). However, knowledge about bats as a potential rabies source was low and not different among main household respondents with and without bat contact. There was no significant difference between main household respondents with and without bat contact regarding history of rabies vaccination and awareness that bats can cause diseases other than rabies. Study participants with bat contact in the 2010 community survey, 2013 community survey, and 2013 bat hunter survey infrequently reported knowledge of any illness as a result of bats or being in a bat cave (1%, 3%, and 0%, respectively) (Appendix 2 Table 1).

Among 170 main household respondents and additional household respondents in the 2013 community survey who participated in the 2013 follow-up survey, 23 (14%) had experienced a febrile illness within 90 days of the February 19, 2013, bat festival (Table 4). Factors such as having had any bat contact within the past 90 days, having touched a bat within the past 90 days, having been bitten by a bat within the past 90 days, having been scratched by a bat within the past 90 days, having participated in the bat festival within the past 90 days, and having entered a bat cave or bat refuge within the past 90 days were not significantly different between those with a febrile illness and those without.

**Table 4:** Characteristics associated with experiencing a febrile illness within 90 days of the bat festival in a community survey of bat exposures, Idanre area, Nigeria, 2013*

Characteristic	Febrile illness within 90 d of bat festival, no. (%), n = 23	No febrile illness within 90 d of bat festival, no. (%), n = 147	p value	OR (95% CI)
Demographics				
Mean age (SD)	47 (18)	43 (17)	0.39	NA
Age range, min–max	18–80	18–89	NA	NA
Median age (interquartile range)	47 (32–65)	38 (30–55)	NA	NA
Age <25 y	2 (9)	18 (12)	0.63	0.68 (0.14–3.27)
Male sex	13 (57)	80 (54)	0.85	1.09 (0.45–2.65)
Education				
Some secondary or above	11 (48)	65 (44)	0.73	1.16 (0.51–2.61)
Completed secondary or above	9 (39)	40 (27)	0.21	1.72 (0.74–4.00)
Household characteristics				
Persons in household				
<5 persons	7 (30)	38 (26)	0.66	1.25 (0.46–3.41)
<10 persons	18 (78)	97 (66)	0.31	1.86 (0.56–6.15)
Main material used to build house				
Adobe/mud	14 (61)	71 (48)	0.29	1.67 (0.65–4.24)
Cement/brick	9 (39)	76 (52)	Referent	Referent
Wood	0	0	NP	NP
Openings present in house that could allow bats to enter	14 (61)	91 (62)	0.93	0.96 (0.38–2.44)
Household with animals†	12 (52)	68 (46)	0.62	1.27 (0.50–3.24)
Household with >1 animal† that has been vaccinated against rabies	2 (17)	6 (9)	0.43	2.07 (0.34–12.64)
Bat contact within past 90 d‡				
Any bat contact	3 (13)	40 (27)	0.15	0.40 (0.11–1.40)
Touched a bat with skin uncovered	3 (13)	40 (27)	0.15	0.40 (0.11–1.40)
Bitten by bat	1 (4)	10 (7)	0.66	0.62 (0.07–5.21)
Scratched by bat	1 (4)	15 (10)	0.39	0.40 (0.05–3.22)
Other bat-related activities within past 90 d				
Participated in bat festival	1 (4)	34 (23)	0.07	0.15 (0.02–1.17)
Entered a bat cave or bat refuge	1 (4)	18 (12)	0.29	0.33 (0.04–2.61)
Prepared a bat as food	7 (30)	57 (39)	0.45	0.69 (0.26–1.82)
Eaten a bat	7 (30)	56 (38)	0.49	0.71 (0.27–1.87)
Knowledge				
Indicated animal bites as mechanism of rabies transmission	13 (57)	78 (53)	0.74	1.15 (0.51–2.62)
Described rabies as severe	13 (57)	84 (57)	0.95	0.98 (0.43–2.23)
Identified bats as a rabies source	1 (4)	3 (2)	0.49	2.18 (0.24–20.11)
Identified dogs as a rabies source	16 (70)	84 (57)	0.26	1.71 (0.67–4.36)
If bitten or scratched by a bat				
Wash wound with soap and water	0	5 (3)	NP	NP
Seek medical care	9 (39)	52 (35)	0.70	1.17 (0.51–2.69)
Seek a traditional healer or pray	2 (9)	5 (3)	0.24	2.70 (0.52–13.97)
Do nothing	9 (39)	69 (47)	0.50	0.73 (0.28–1.85)
If bitten by a potentially rabid animal				
Wash wound with soap and water	0	1 (1)	NP	NP
Seek medical care	16 (70)	92 (63)	0.53	1.37 (0.51–3.64)
Seek a traditional healer or pray	3 (13)	4 (3)	0.03	5.36 (1.17–24.48)
Do nothing	3 (13)	33 (22)	0.32	0.52 (0.14–1.89)
History of rabies vaccination	1 (4)	1 (1)	0.19	6.64 (0.39–111.64)
Aware that bats can cause disease other than rabies	3 (13)	6 (4)	0.08	3.53 (0.86–14.40)
Know of reports of illness as a result of bats or being in bat cave	2 (9)	1 (1)	0.03	13.90 (1.25–154.63)

*NA, not applicable or not calculated; NP, logistic regression could not be performed due to zero cells; OR, odds ratio. †Pet or livestock. ‡Bat contact was defined as having touched a bat, having been bitten by a bat, or having been scratched by a bat.

Among 18 participants from the 2013 bat hunter survey who participated in the 2013 follow-up survey, 7 (39%) had experienced a febrile illness within 90 days of the February 19, 2013, bat festival. Mean age was significantly higher among those with a febrile illness compared with those without (61 years vs. 49 years; p = 0.048). The odds of having entered a bat cave or bat refuge within the past 90 days was significantly higher among those without a febrile illness compared with those with a febrile illness (p = 0.03). There were no other significant differences between those with a febrile illness and those without when analyzing the same characteristics (Table 4).

Of all study participants who underwent serologic testing, only 2 had lyssavirus neutralizing antibodies, both against rabies virus (Appendix 2 Table 2). Both denied recent encephalitis-like illness or having ever received rabies vaccine, but 1 reported prior bat contact. One of these respondents underwent repeat serologic testing for rabies virus neutralizing antibodies during the 2013 follow-up survey, and rabies virus neutralizing antibodies were still detectable.

We sampled 211 bats: 120 bats during September 2010 (112 *Rousettus aegyptiacus*, 8 *Hipposideros gigas*) and 91 during February 2013 (all *R. aegyptiacus*); none demonstrated clinical illness at time of capture. No *R. aegyptiacus* bats had neutralizing antibodies to Duvenhage virus; >50% had neutralizing antibodies to Lagos bat virus, Shimoni bat virus, and Mokola virus; and 1 had neutralizing antibodies to Ikoma lyssavirus (Table 5; Appendix 2 Table 3). Lyssavirus antigens were not detected in brain specimens from any of the 211 bats.

**Table 5:** Summary of serologic testing results for lyssavirus antibodies among *Rousettus aegyptiacus* bats roosting in caves used in a bat festival, Idanre area, Nigeria, 2010 and 2013*

Lyssavirus type (species)	Duvenhage virus (South Africa, 1970)	Lagos bat virus (lineage B, Nigeria, 1956)	Shimoni bat virus (Kenya, 2009)	Mokola virus (South Africa, 1998)	Ikoma lyssavirus (Tanzania, 2009)
Lyssavirus phylogroup	I	II	II	II	Undetermined
Year	2013	2010, 2013	2013	2013	2013
No. bats tested	67	169	60	62	64
No. (%) bats with detectable neutralizing antibodies	0	89 (53)	30 (50)	37 (60)	1 (2)

*A total of 211 bats were collected: 120 bats during September 2010 (112 *Rousettus aegyptiacus*, 8 *Hipposideros gigas*) and 91 during February 2013 (all *R. aegyptiacus*). This table displays only data on serologic testing for lyssaviruses among *R. aegyptiacus* bats; serum specimens were not available for all *R. aegyptiacus* bats.

## Discussion

The occurrence of purposeful human interactions with bats, such as hunting for food (e.g., bushmeat), has been identified in several parts of the world and can pose a risk to human health through spillover of zoonotic pathogens from bats to humans (*29*–*31*). We therefore investigated bat and lyssavirus exposures among humans in an area of Nigeria that celebrates a biannual bat festival. Overall, we found that persons who interact with bats in this area are likely at risk for phylogroup II lyssavirus exposures, and public health precautions are warranted.

Although nearly two thirds of households enrolled in our study had >1 household members who had ever had bat contact, only about one quarter of households reported having >1 household members who had ever participated in the bat festival. This finding strongly suggests that a sizable proportion of the human population in the area has had bat exposures unrelated to the bat festival. Furthermore, 10% of persons living within households enrolled in our community surveys had previously had bat contact and 2% had been bitten by a bat. We do not know whether the bat contact and bat bites among these persons are related to participation in the bat festival or to interactions with bats from the festival caves. Because entry into the festival caves is allowed only during the bat festivals, we suspect that many of these persons have had interactions with bats that are not from the festival caves. Regardless, these person-level data on the prevalence of bat contact and bat bites are likely an underestimate of the true prevalence of bat contact and bat bites in the area; persons with a history of bat interactions might not have been available or were not referred by other household members so they were not enrolled in the study, or persons who have had such bat interactions might have failed to report them when responding to the survey.

We also found strong serologic evidence that lyssaviruses circulate among bats in the festival caves. We found neutralizing antibodies to Lagos bat virus, Shimoni bat virus, and Mokola virus in >50% of bats, which is higher than in some prior reports (*17*,*32*–*34*). All 3 of these lyssaviruses belong to phylogroup II. We did not detect lyssavirus antigen in brains of any seropositive bat that we captured, suggesting that these bats survived past exposure to a phylogroup II lyssavirus. We cannot be sure which phylogroup II lyssavirus predominantly circulates in this bat population, given potential serologic cross reactivity and because we did not isolate any lyssavirus from bats. However, we suspect Lagos bat virus because it has been documented in *R. aegyptiacus* bats before and because it was first isolated in a fruit bat in Nigeria, although we cannot rule out the possibility that a yet uncharacterized phylogroup II lyssavirus circulates among these bats (*18*,*35*).

Although some respondents reported a febrile illness after the 2013 bat festival, this finding was not associated with having recent bat contact or recent participation in the bat festival. We recommend caution in interpreting these findings. A variety of bat species, including *R. aegyptiacus*, which we identified in the festival caves, are known reservoirs for a range of potential pathogens, including filoviruses and coronaviruses (*18*,*22*,*36*,*37*). It is therefore plausible that at least some zoonotic pathogens are present in bats residing in the festival caves and that these pathogens can spill over into humans (*16*). Furthermore, the data we present on febrile illness are a snapshot from 2013, and given that excretion of virus in bats can be episodic, the risk of batborne infections may vary over time (*23*).

We did not find neutralizing antibodies to lyssaviruses in any person in the study, other than 2 persons who had neutralizing antibodies to rabies virus, perhaps reflecting prior rabies vaccination that was not recalled during the survey or abortive infection from bites of rabid dogs (*5*). Thus, we found no evidence of abortive phylogroup II lyssavirus infections among humans in this study, despite the high prevalence of neutralizing antibodies to phylogroup II lyssaviruses among bats in the festival caves and that many persons in the area frequently interact with bats. This result is perhaps not surprising. First, as previously explained, we suspect that many interactions with bats among the population are unrelated to the bat festival and unrelated to bats from the festival caves (although bat hunters who participated in the 2013 bat hunter survey, by definition, would have had interaction with bats from the festival caves). The data we present on the prevalence of neutralizing antibodies to phylogroup II lyssaviruses among bats are specific to bats from the festival caves and cannot be generalized to other bat populations in the area; the prevalence of these antibodies in other bat populations with which humans also interact might be lower than that for bats from the festival caves. Second, in the Amazon, where abortive lyssavirus infections have been documented, humans likely experience bat bites on a more continuous basis because of the predatory nature of vampire bats (*5*). In contrast, the bat festival in this part of Nigeria occurs at discrete times, leading to a lower frequency of bat bites and thus lower risk of lyssavirus exposure. Finally, the dates of the bat festivals vary each year and are determined based on traditional wisdom. Whether the bat festival timing, as determined by cultural leaders, implicitly accounts for periods of lower risk of batborne infections to festival participants warrants further investigation by an interdisciplinary team of biologists and anthropologists (*23*).

Our study has limitations. Accurate information on the distribution of communities in the area was limited, making it unclear whether persons we enrolled are representative of the area. We did not use a strict definition for febrile illness, nor could we verify the occurrence of a febrile illness; rather, we relied on retrospective, subjective reports. Our study did not have a robust method of identifying encephalitis-like illness and deaths that occurred between the initial data collection in 2013 and the 2013 follow-up survey, and we do not know what happened to participants who could not be located for the follow-up survey. Thus, we cannot draw conclusions on the ability of the predominant phylogroup II lyssavirus that circulates among bats in the festival caves to cause productive lyssavirus infections (rabies) in humans.

Emerging infectious diseases are on the rise around the world; most originate from animals (*38*). Although the source of the 2014–2016 Ebola outbreak remains unknown, it may have begun with a single spillover event involving initial bat contact (*39*), which underscores the health risks of interacting with bats without appropriate precautions. If we assume that the households we enrolled are representative of the Idanre area, then this part of Nigeria has high rates of bat contact and is at high risk for bat-related zoonoses. We therefore recommend that officials strengthen health security in the Idanre area, recognizing that an approach that bans hunting and consumption of bats is unlikely to be effective. Rather, a more productive approach will focus on harm reduction and community engagement. Specific recommendations include educating the population, particularly persons who participate in high-risk bat-related activities, about the health risks associated with bats and the ecosystem benefits provided by bats; providing preexposure prophylaxis for rabies and possibly other batborne disease (potentially even Ebola) for persons who participate in high-risk bat-related activities; and developing surveillance and outbreak response capacity in the area for syndromes such as febrile illness, encephalitis, and hemorrhagic fevers.

Appendix 1 Questionnaires used for study of bat and lyssavirus exposure among humans in area that celebrates a bat festival, Nigeria, 2010 and 2013.

Appendix 2 Additional information about bat and lyssavirus exposure among humans in area that celebrates a bat festival, Nigeria, 2010 and 2013.
