# Supplementary material for: Bat and Lyssavirus Exposure among Humans in Area that Celebrates Bat Festival, Nigeria, 2010 and 2013
Source: Emerg Infect Dis. 2020 Jul;26(7):1399–408. doi: 10.3201/eid2607.191016 (PMC7323560; doi:10.3201/eid2607.191016)
Supplement: Appendix 1 — Questionnaires used for study of bat and lyssavirus exposure among humans in area that celebrates a bat festival, Nigeria, 2010 and 2013. [file 19-1016-Techapp-s1.pdf]

Article DOI: https://doi.org/10.3201/eid2607.191016

Bat and Lyssavirus Exposure among Humans in Area that Celebrates Bat Festival, Nigeria, 2010 and 2013

Appendix 1

Questionnaires Used for Surveys about Bat Exposure

The following pages show a set of questionnaires used to survey community members and bat hunters regarding bat exposures in Idanre, Nigeria, in 2010 and 2013:

- Two community surveys conducted during September 26–28, 2010 (9–11 days after the September 17, 2010, bat festival took place), and March 2–March 6, 2013 (11–15 days after the February 19, 2013, bat festival took place);
- A survey of bat hunters conducted on March 6, 2013;
- A follow-up survey of subjects who participated in the 2013 community survey or 2013 bat hunter survey, conducted during May 14–19, 2013 (85–90 days after the February 19, 2013, bat festival took place).

1. Questionnaire used in two community surveys and a bat hunter survey of bat exposures — Idanre, Nigeria, 2010 and 2013

A. HOUSEHOLD INFORMATION

1) Household ID Number

2) GPS

3) Municipality

4) Community

[Section A. Administer to adult (18 years and older) present that attended door call (main responder).

Hello. My name is ____________________ and I am working with the <insert appropriate agency affiliation>.

We are conducting a survey to improve our understanding of the knowledge, attitudes, and practices of people in Idanre local government of Ondo State, who come in close contact with bats or places where bats live, like caves. We would like to ask for a few minutes of your time, around 40 minutes, to ask you about your experiences with bats and your knowledge about certain diseases. Your answers to the following questions are completely voluntary and will be kept confidential. Do you have time now? (If NO, "Thank you for your time.")

5) Is there someone in the house that can respond to the interview?

Yes

No

Not applicable, because interview is being conducted on a person returning from a cave

6)

6.1) Consent obtained?

Yes

No

6.2) If consent was NOT obtained

6.2.1) Reason for declining

Not interested

No time

Fear of participating

Not capable of consenting

Language barrier

Other

6.2.2) If the reason is "other"
6.2.2.1) Specify the reason for denying consent

6.2.3) Interviewer name: (First Name, First Family Name, Second Family name)

6.3) If consent obtained

Please think carefully about each question, and answer as well as you can. You can choose not to answer any of the questions.

NOTE to INTERVIEWER: Do not read the answer choices, unless otherwise indicated. Circle the choice that best represents the interviewee's answer.

6.3.1) How many people live in this home?

6.3.2) How many are females living in this home?

6.3.3) How many children of ages 0-5 live in this house?

6.3.4) How many children of ages 6-12 live in this house?

6.3.5) How many children of ages 13-17 live in this house?

6.3.6) About the house, which is the main material used to build the house?
Brick
Adobe
Wood
Canes
Cement Block
Cement / Concrete
Other

6.3.7) If the house is made of another material
6.3.7.1) Specify the other housing material

6.3.8) Does the house have windows/doors that close and prevent bat entry? (Check all that apply)
There are open windows
Windows can close completely
Doors can close completely
There are windows or doors that close incompletely and allow entry of bats
There are large openings in the walls for ventilation never closed

No openings
Other: ______________
Don’t know
Declined to answer

6.3.9) Do you own animals as either pets or livestock?

Yes
No
Don't know
Declined to answer

6.3.10) If you own animals as either pets or livestock
6.3.10.1) Do you know or have you seen if your domestic animals (pets/cattle/pigs) have been bitten by bats?
Yes
No

6.3.10.2) If your domestic animals have been bitten by bats
6.3.10.2.1) Which of your animals have been bitten by bats? (Select all that apply?)
Cows
Goats
Sheep
Pigs
Horses
Dogs
Cats
Chicken
Other

6.3.10.2.2) If the bitten animal is "Other"
6.3.10.2.2.1) Specify the animals that have been bitten by bats

6.3.10.3) Do you do anything to avoid your animals/pets being bitten by bats?
Nothing
Lights on where animals sleep
Barriers (nets, close doors)
Burn herbs
Apply oil/chemicals to animals
Hunt bats
Blankets
Garlic

Other

6.3.10.4) If answered OTHER as something that is done to avoid your animals/pets being bitten by bats

6.3.10.4.1) Specify what other thing is done to avoid your animals/pets being bitten by bats

6.3.10.5) Are one or more of your animals vaccinated against rabies?

Yes
No
Don't know
Declined to answer

6.3.10.6) Have any of your animals been sick or died due to bats?

Yes
No
Don't know
Declined to answer

6.3.10.7) If any of your animals have been sick or died due to an illness that you believe may have been caused by bats?

**Complet e for each species:**	**Questions about animal sickness**	**What were their signs? (tick all that apply)**	**Questions about animal death**
A) Goats	a) How many got sick?<br>☐ 0 ☐ 1<br>☐ 2 ☐ 3+<br>☐ D/K ☐ Declined	c) What were their signs? (tick all that apply)<br>☐ Not moving much/hiding ☐ Problems walking	d) How many died?<br>☐ 0 ☐ 1<br>☐ 2 ☐ 3+

	b) Is/are the animal recovered from the symptoms? (yes, no, unsure, declined)	☐ Not eating well ☐ Vomiting ☐ Diarrhea<br>☐ Foaming at mouth/salivation<br>☐ Bellowing/crying ☐ Trembling or twitching<br>☐ Behavior change (more quiet/more aggressive)	☐ D/K ☐ Declined<br>e) Was/were the animal(s) slaughtered and eaten or sold for food? (yes, no, some, unsure, declined)<br>f) Was/were any of the animals sick before they

		☐ Coughing ☐ Sneezing ☐ Runny nose<br>☐ Problems breathing ☐ Convulsions<br>☐ Still birth ☐ Suddenly died<br>☐ Other (specify):	were slaughtered or sold? (yes, no, some, unsure, declined)
B) Sheep	a) How many got sick?<br>☐ 0 ☐ 1<br>☐ 2 ☐ 3+	c) What were their signs? (tick all that apply)	d) How many died?<br>☐ 0 ☐ 1<br>☐ 2 ☐ 3+

	☐ D/K ☐ Declined<br><br>b) Is/are the animal recovered from the symptoms? (yes, no, unsure, declined)	☐ Not moving much/hiding ☐ Problems walking<br>☐ Not eating well ☐ Vomiting ☐ Diarrhea<br>☐ Foaming at mouth/salivation<br>☐ Bellowing/crying ☐ Trembling or twitching	☐ D/K ☐ Declined<br><br>e) Was/were the animal(s) slaughtered and eaten or sold for food? (yes, no, some, unsure, declined)<br><br>f) Was/were any of the animals sick before they

		☐ Behavior change (more quiet/more aggressive)<br>☐ Coughing ☐ Sneezing ☐ Runny nose<br>☐ Problems breathing ☐ Convulsions<br>☐ Still birth ☐ Suddenly died<br>☐ Other (specify):	were slaughtered or sold? (yes, no, some, unsure, declined)

C) Cows	a) How many got sick?<br>☐ 0 ☐ 1<br>☐ 2 ☐ 3+<br>☐ D/K ☐ Declined<br><br>b) Is/are the animal recovered from the	c) What were their signs? (tick all that apply)<br>☐ Not moving much/hiding ☐ Problems walking<br>☐ Not eating well ☐ Vomiting ☐ Diarrhea<br>☐ Foaming at mouth/salivation	d) How many died?<br>☐ 0 ☐ 1<br>☐ 2 ☐ 3+<br>☐ D/K ☐ Declined<br><br>e) Was/were the animal(s) slaughtered and eaten or sold

	symptoms? (yes, no, unsure, declined)	☐ Bellowing/crying ☐ Trembling or twitching<br>☐ Behavior change (more quiet/more aggressive)<br>☐ Coughing ☐ Sneezing ☐ Runny nose<br>☐ Problems breathing ☐ Convulsions<br>☐ Still birth ☐ Suddenly died	for food? (yes, no, some, unsure, declined)<br>f) Was/were any of the animals sick before they were slaughtered or sold? (yes, no, some, unsure, declined)

		☐ Other (specify):	
D) Pigs	a) How many got sick?<br>☐ 0 ☐ 1<br>☐ 2 ☐ 3+<br>☐ D/K ☐ Declined	c) What were their signs? (tick all that apply)<br>☐ Not moving much/hiding ☐ Problems walking<br>☐ Not eating well ☐ Vomiting ☐ Diarrhea	d) How many died?<br>☐ 0 ☐ 1<br>☐ 2 ☐ 3+<br>☐ D/K ☐ Declined

	b) Is/are the animal recovered from the symptoms? (yes, no, unsure, declined)	☐ Foaming at mouth/salivation<br>☐ Bellowing/crying ☐ Trembling or twitching<br>☐ Behavior change (more quiet/more aggressive)<br>☐ Coughing ☐ Sneezing ☐ Runny nose<br>☐ Problems breathing ☐ Convulsions	e) Was/were the animal(s) slaughtered and eaten or sold for food? (yes, no, some, unsure, declined)<br>f) Was/were any of the animals sick before they were slaughtered or sold?

		☐ Still birth ☐ Suddenly died<br>☐ Other (specify):	(yes, no, some, unsure, declined)
E) H orses	a) How many got sick?<br>☐ 0 ☐ 1<br>☐ 2 ☐ 3+<br>☐ D/K ☐ Declined	c) What were their signs? (tick all that apply)<br>☐ Not moving much/hiding ☐ Problems walking<br>☐ Not eating well ☐ Vomiting ☐ Diarrhea	d) How many died?<br>☐ 0 ☐ 1<br>☐ 2 ☐ 3+<br>☐ D/K ☐ Declined

Household ID#

	b) Is/are the animal recovered from the symptoms? (yes, no, unsure, declined)	☐ Foaming at mouth/salivation<br>☐ Bellowing/crying ☐ Trembling or twitching<br>☐ Behavior change (more quiet/more aggressive)<br>☐ Coughing ☐ Sneezing ☐ Runny nose<br>☐ Problems breathing ☐ Convulsions<br>☐ Still birth ☐ Suddenly died<br>☐ Other (specify):	e) Was/were the animal(s) slaughtered and eaten or sold for food? (yes, no, some, unsure, declined)<br>f) Was/were any of the animals sick before they were slaughtered or sold? (yes, no, some, unsure, declined)
F) Dogs	a) How many got sick?<br>☐ 0 ☐ 1<br>☐ 2 ☐ 3+<br>☐ D/K ☐ Declined<br>b) Is/are the animal recovered from the symptoms? (yes, no, unsure, declined)	c) What were their signs? (tick all that apply)<br>☐ Not moving much/hiding ☐ Problems walking<br>☐ Not eating well ☐ Vomiting ☐ Diarrhea<br>☐ Foaming at mouth/salivation<br>☐ Bellowing/crying ☐ Trembling or twitching<br>☐ Behavior change (more quiet/more aggressive)<br>☐ Coughing ☐ Sneezing ☐ Runny nose<br>☐ Problems breathing ☐ Convulsions<br>☐ Still birth ☐ Suddenly died<br>☐ Other (specify):	d) How many died?<br>☐ 0 ☐ 1<br>☐ 2 ☐ 3+<br>☐ D/K ☐ Declined<br>e) Was/were the animal(s) slaughtered and eaten or sold for food? (yes, no, some, unsure, declined)<br>f) Was/were any of the animals sick before they were slaughtered or sold? (yes, no, some, unsure, declined)

7) Are there any dogs in your house?
    Yes
    No
7.1) If there are dogs in the house
7.1.1) How many dogs:______
7.1.2) How many female dogs: ___________
7.1.3) How many male dogs: _____________
7.1.4) What is the age of the oldest dog in years?: ___
7.1.5) What is the age of the youngest dog?:________
7.1.6) Are the dogs in your house vaccinated against rabies?:
    Yes, all of them
    Yes, but only some of them
    No, none of them are vaccinated
    Don't know
    Decline to answer
    7.1.6.1) If yes, when was the last vaccination:
        Date: _________

7.1.7) Have any of the dogs in the house bitten anybody?
Yes
No
Don’t know
Decline to answer

7.1.8) Have any of the dogs in the house bitten any of your other animals?
Yes
No
Don’t know
Decline to answer

B. HOUSEHOLD RESPONDENT INFORMATION

1A) Sample ID

1B) Name: First name, First Family name, Second Family name

1B.1) What is your home address?

1B.2) What is your mobile phone number? (if no mobile phone, get home phone instead)

1C) Where do you live when this bat festival does not take place?
Idanre local government, Ondo State, Nigeria
Ondo state (but not Idanre local government), Nigeria
A state other than Ondo State, but in Nigeria
An African country other than Nigeria
A country outside of Africa

1D) If you do not live in Ondo State, what brought you to Ondo State at this time? (circle all that apply)
To participate in the bat festival
For vacation but not specifically for the bat festival
To see family and friends
For work/to make money
Other

1D.1) If other, please specify why:

1E) Did you participate in a survey like this about bats and the bat festival nearly three years ago?
Yes
No
Declined to answer

2) How old are you?

3) Gender

Male
Female

4) What is the last level of schooling you have completed? (Note to interviewer: Read all but last two options to participant.)

None
Started primary
Completed primary
Started basic/Junior Secondary School (JSS)
Completed basic/Junior Secondary School (JSS)
Started secondary/Senior Secondary School (SSS)
Completed secondary/Senior Secondary School (SSS)
Started higher education
Completed higher education
Not sure
Declined to answer

5) How many years have you lived in this house?
Less than one year
One year
More than one year

6) If you have lived in this house more than one year
6.1) how many years

7) How many years of experience do you have working/living with or near bats?
None
5 or less
6-15
16-25
> 25
Don't know
Declined to answer

8) What activities do you engage in that regularly puts you in contact with bats? (check all that applies) (Note to interviewer: Read all but last two options to participant.)

Bat hunting
Participation in bat festival
Preparing bats for consumption
Farming
Hunting
Nightwatchman
Other

None
Declined to answer

9) If the activity is Other
9.1)

9.2) Have you ever participated in the bat festival (if yes, go to the next question, and if no, skip to question 10)
Yes
No
Declined to answer

9.2.1) How many times or often do you participate in the bat festival?
Once a year
Twice a year
Don’t know
Declined to answer

9.2.2) How many years have you participated in the bat festival?

Less than 2 years
2 years to less than 5 years
5 years to less than 20 years
20 years and more
Do not know
Declined to answer

9.2.3) what role do you play during the bat festival? (tick all that concerns)
Bat hunting
Selling of bats
Preparation of bats for food/consumption
Dancer, singer
Spiritual activities
Standby watcher
Don’t know
Decline to answer
Other ____________

10) Have you been inside of a bat cave or bat refuge (trees, abandoned house, bridge, etc.)?
Yes
No
Don't know
Declined to answer

11) If you have been inside of a bat cave or refuge

11.1) How often do you enter bat caves or bat refuge? (Note to interviewer: Read all but last two options to participant.)

once per year or less
2-4 times per year
5 times or more per year
Don’t know
Declined to answer

11.2) When was the last time you entered a bat cave or bat refuge? (Note to interviewer: Read all but last two options to participant.)

< 1 month ago
1 to 6 months ago
6-12 months ago
More than 12 months ago
Don’t know
Declined to answer

12) Have you ever touched a live bat with your skin uncovered?

Yes
No
Don't know
Declined to answer

13) If you have ever touched a live bat with your skin uncovered

13.1) How often do you touch bats? (Note to interviewer: Read all but last two options to participant.)

once per year or less
2-4 times per year
5 times or more per year
Don’t know
Declined to answer

13.2) When was the last time you touched a bat with your skin uncovered? (Note to interviewer: Read all but last two options to participant.)

< 1 month ago
1 to 6 months ago
6-12 months ago
More than 12 months ago
Don’t know

Declined to answer

14) Have you ever been scratched by a bat, to your knowledge?
Yes
No
Don't know
Declined to answer

15) If has been scratched by a bat
15.1) How often are you scratched by bats? (Note to interviewer: Read all but last two options to participant.)

once per year or less
2-4 times per year
5 times or more per year
Don't know
Declined to answer

15.2) When was the last time you were scratched by a bat? (Note to interviewer: Read all but last two options to participant.)

< 1 month ago
1 to 6 months ago
6-12 months ago
More than 12 months ago
Don't know
Declined to answer

16) Have you ever been bitten by a bat, to your knowledge?
Yes
No
Don't know
Declined to answer

17) If you have been bitten by a bat
17.1) How often are you bitten by bats? (Note to interviewer: Read all but last two options to participant.)

once per year or less
2-4 times per year
5 times or more per year
Don't know
Declined to answer

17.2) When was the last time you were bitten by a bat? (Note to interviewer: Read all but last two options to participant.)

< 1 month ago
1 to 6 months ago
6-12 months ago
More than 12 months ago
Don’t know
Declined to answer

18) Have you ever prepared a bat as food?
Yes
No
Don't know
Declined to answer

19) If you ever prepared a bat as food
19.1) How often do you prepare them for eating (Note to interviewer: Read all but last two options to participant.)

once per year or less
2-4 times per year
5 times or more per year
Don’t know
Declined to answer

19.2) When was the last time you prepared one for eating? (Note to interviewer: Read all but last two options to participant.)

< 1 month ago
1 to 6 months ago
6-12 months ago
More than 12 months ago
Don’t know
Declined to answer

20) Have you ever eaten a bat?
Yes
No
Don't know
Declined to answer

21) If you have ever eaten a bat
21.1) How often do you eat bats? (Note to interviewer: Read all but last two options to participant.) (Note to interviewer: Read all but last two options to participant.)

once per year or less
2-4 times per year
5 times or more per year
Don’t know
Declined to answer

21.2) When was the last time you ate a bat? (Note to interviewer: Read all but last two options to participant.)

< 1 month ago
1 to 6 months ago
6-12 months ago
More than 12 months ago
Don’t know
Declined to answer

22) What kinds of bats do you most frequently observe or have had contact with? (Note to interviewer: Read all but last two options to participant.)

Fruit-eating bats
Insect-eating bats
Vampire bats
Multiple types
Other
Don’t know
Declined to answer

23) If the kind of bat is "Other"
23.1) Specify the other type of bat

24) Do you or your family do something to avoid bat bites in the house?
Nothing
Use mosquito net
Prevent entry of bat in the house
Increase number of cats
Increase the number of cattle/pigs to be bitten
Destroy bat refuges/kill bats
Pray/consult the gods
Declined to answer
Other

25) If answered OTHER as the type of action taken to avoid bites in the house
25.1) Specify what you and your family does to avoid bat bites in the house

26) How much do you know about rabies? (Note to interviewer: Read all but last two options to participant.)

Little to none
Basic
Extensive
Declined to answer

27) How dangerous is rabies?
Very Severe
Mild or moderate
Don't know
Declined to answer

28) How do people get infected with rabies? (Note to interviewer: Read all but last two options to participant.)

Animal bite
Animal scratch or lick
Touching an animal
Eating an animal
Other
Don't know
Declined to answer

29) If the way people are infected with rabies is "Other"
29.1) Specify the way people are infected by rabies

30) What animals can be infected with rabies? (check all that apply) (Note to interviewer: Read all but last two options to participant.)

Bats
Dogs
Cats
Horses
Livestock
Wild mammals (not bats)
Other
Don't know
Declined to answer

31) If the animals are potentially infected with rabies are "Other"
31.1) Specify which other animals could be infected with rabies

32) What would you do if you were bitten or scratched by a bat? (Note to interviewer: Read all but last two options to participant. Select all that apply.)

Nothing

Wash wound with soap and water
Call a doctor for advice
Call or visit a traditional healer
Seek medical care at a hospital, clinic or health post
Seek rabies PEP (rabies vaccines)
Have bat tested for rabies (or other diseases)
Other
Don’t know
Declined to answer

33) If the action that you would take is Other
33.1) Specify the other action that would be taken

34) Do you think there is any time of the year in which bats attack more animals or people?
No, it is the same all year round
Yes, rainy season (April-October)
Yes, dry season (November-April)
Don't know
Declined to answer

35) If someone has been bitten by an animal potentially infected with rabies what should that person do? (Check all that apply)
Nothing
Wash wound with soap and water
Call a doctor for advice
Call or visit a traditional healer
Seek medical care at a hospital or clinic
Seek rabies post-exposure prophylaxis (rabies vaccines)
Check animal’s vaccination history
Observe animal for a period of time to see if it becomes rabid
Have animal tested for rabies
Kill animal
Other
Don't know
Declined to answer

36) If the action is "Other"
36.1) Specify the other action that should be done if someone has been bitten by an animal that might be infected by rabies

37) Have you ever been vaccinated against rabies?
Yes
No
Don't know

Declined to answer

38) If you have ever been vaccinated against rabies

38.1) What was the reason you were vaccinated against rabies?

Post-exposure prophylaxis
Pre-exposure prophylaxis
Have received PreP and PEP
Don't know
Declined to answer

38.2) If you have received rabies vaccination after being bitten or scratched by an animal bite, what animal or animals were responsible for the incident? (check all that apply)

Bats
Dogs
Cats
Horses
Livestock
Wild mammals (not bats)
Others
Don't know
Declined to answer
Did not receive PEP

38.3) If received a vaccination after being bitten by an OTHER animal

38.3.1) Specify the other animal that bit you

39) Are you aware if there are any other diseases that humans can get from bats? (NOTE: any disease mentioned means "yes')

Yes
No
Don't know
Declined to answer

40) Have you or anyone you know ever experienced an illness that you believe may have been caused by bats or being in a bat cave?

Yes
No
Don't know
Declined to answer

41) If you or anyone you know ever experienced an illness that you believe may have been caused by bats or being in a bat cave

41.1) What were the symptoms? (Check all that apply) (Note to interviewer: If respondent doesn't indicate that the person(s) recovered, ask if they died from illness. If answer is yes, circle death as a symptom. If respondent knows of more than one person affected—

including but necessarily themselves—and symptoms mentioned are a composite, circle "multiple persons".)

Skin rash/discoloration/ infection
Unusual bleeding (e.g. from nose/mouth)
Fever
Cough
Sneezing
Runny nose
Chest congestion
Muscle pain
Difficulty breathing
Headache
Convulsions
Altered mental state (dementia)
Unconsciousness/coma
Muscle weakness/paralysis
Vomiting or diarrhea or stomach cramps
Miscarriage/stillbirth
Death
Multiple persons
Other
Don't know
Declined to answer

41.2) If the symptoms is "Other"
41.2.1) Specify the other symptom

41.2.2) Are you or the person you know that presented symptoms caused by a bat recovered?
Yes
No
Don't know
Decline to answer

42) We would like to take a sample of your blood. Will you allow us to take a sample?

Yes
No
42.2 Was blood sampled obtained?
Yes
No
42.3. IF blood sample was not obtained, why not?
Did not consent for blood
Was not able to get blood

Other:________________

42.4) Will you allow us to return in 6-8 weeks to ask you some more questions? YES/ NO

43) Has anyone from your family or living here had been in contact, bitten, scratched, eaten, or had touched a bat?

Yes

No

C. PARTICIPANTS WITH BAT EXPOSURE (ADDITIONAL TO RESPONDENT)

Additional Participant

Interviewer name: (First Name, First Family Name, Second Family name)_

1A) Sample ID

1B) Name: First name, first family name, second family name

1C) Household ID: ___________________

2) How old are you?

3) If at least 18 years old or mature minors

3.1) Consent obtained? (If yes, go to question 3.2)

Yes

No

3.2) What is your mobile phone number? (if no mobile phone, get home phone instead)

4) If less than 18 years old

4.1) Parental permission obtained?

Yes

No

4.2) Children between 7 and 17 years [Interviewer: parents will answer the survey when child < 9 years of age but child age 9 years and older will answer survey directly]

4.2.1) Child assent obtained?

Yes

No

5) If consent obtained (and assent if applicable)

5.1) Interviewer: who is being interviewed:

- ○ Self
- ○ Parent/guardian

5.2) Gender

Male
Female

5.2.1) Did you participate in a survey like this about bats and the bat festival nearly three years ago?

Yes
No
Declined to answer

5.3) What is the last level of schooling you have completed?

None
Started primary
Completed primary
Started basic/Junior Secondary School (JSS)
Completed basic/Junior Secondary School (JSS)
Started secondary/Senior Secondary School (SSS)
Completed secondary/Senior Secondary School (SSS)
Started higher education
Completed higher education
Not sure
Declined to answer

5.4) How many years have you lived in this house?

Less than one year
One year
More than one year

5.4.1) Where do you live when this bat festival does not take place?

Idanre local government, Ondo State, Nigeria
Ondo state (but not Idanre local government), Nigeria
A state other than Ondo State, but in Nigeria
An African country other than Nigeria
A country outside of Africa

5.4.2) If you do not live in Ondo State, what brought you to Ondo State at this time? (circle all that apply)

To participate in the bat festival
For vacation but not specifically for the bat festival
To see family and friends
For work/to make money
Other

5.4.2.1) If other, please specify why:

5.5) If you have lived in this house more than one year
5.5.1) how many years

5.6) How many years of experience do you have working/living with or near bats? (Note to interviewer: Read all but last two options to participant.)

None
5 or less
6-15
16-25
> 25
Don't know
Declined to answer

5.7) What activities do you engage in that regularly puts you in contact with bats? (check all that applies) (Note to interviewer: Read all but last option to participant.)

Bat hunting
Participation in bat festival
Preparing bats for consumption
Farming
Hunting
Nightwatchman
Other
None
Declined to answer

5.8) If the activity is Other
5.8.1)

5.8.2) Have you ever participated in the bat festival (if yes, go to the next question, and if no, skip to question 5.9)
Yes
No
Declined to answer

5.8.3) How many times or often do you participate in the bat festival?
Once a year
Twice a year
Don’t know
Declined to answer

5.8.4) How many years have you participated in the bat festival?

Less than 2 years
2 years to less than 5 years
5 years to less than 20 years
20 years and more
Do not know
Declined to answer

5.8.5) what role do you play during the bat festival? (tick all that concerns)
Bat hunting
Selling of bats
Preparation of bats for food/consumption
Dancer, singer
Spiritual activities
Standby watcher
Don't know
Decline to answer
Other ____________

5.9) Have you been inside of a bat cave or bat refuge (trees, abandoned house, bridge, etc.)?
Yes
No
Don't know
Declined to answer

5.10) If you have been inside of a bat cave or refuge
5.10.1) How often do you enter bat caves or bat refuge? (Note to interviewer: Read all but last two options to participant.)

once per year or less
2-4 times per year
5 times or more per year
Don't know
Declined to answer

5.10.2) When was the last time you entered a bat cave or bat refuge? (Note to interviewer: Read all but last two options to participant.)

< 1 month ago
1 to 6 months ago
6-12 months ago
More than 12 months ago
Don't know

Declined to answer

5.11) Have you ever touched a live bat with your skin uncovered?
Yes
No
Don't know
Declined to answer

5.12) If you have ever touched a live bat with your skin uncovered
5.12.1) How often do you touch bats? (Note to interviewer: Read all but last two options to participant.)
once per year or less
2-4 times per year
5 times or more per year
Don’t know
Declined to answer

5.12.2) When was the last time you touched a bat?
< 1 month ago
1 to 6 months ago
6-12 months ago
More than 12 months ago
Don’t know
Declined to answer

5.13) Have you ever been scratched by a bat, to your knowledge?
Yes
No
Don't know
Declined to answer

5.14) If has been scratched by a bat
5.14.1) How often are you scratched by bats? (Note to interviewer: Read all but last two options to participant.)
once per year or less
2-4 times per year
5 times or more per year
Don’t know
Declined to answer

5.14.2) When was the last time you were scratched by a bat?
< 1 month ago
1 to 6 months ago
6-12 months ago
More than 12 months ago

Don’t know
Declined to answer

5.15) Have you ever been bitten by a bat, to your knowledge?
Yes
No
Don't know
Declined to answer

5.16) If you have been bitten by a bat
5.16.1) How often are you bitten by bats? (Note to interviewer: Read all but last two options to participant.)
once per year or less
2-4 times per year
5 times or more per year
Don’t know
Declined to answer

5.16.2) When was the last time you were bitten by a bat? (Note to interviewer: Read all but last two options to participant.)

< 1 month ago
1 to 6 months ago
6-12 months ago
More than 12 months ago
Don’t know
Declined to answer

5.17) Have you ever prepared a bat as food?
Yes
No
Don't know
Declined to answer

5.18) If you have ever prepared a bat as food
5.18.1) How often do you prepare them for eating? (Note to interviewer: Read all but last two options to participant.)
once per year or less
2-4 times per year
5 times or more per year
Don’t know
Declined to answer

5.18.2) When was the last time you prepared a bat for eating? (Note to interviewer: Read all but last two options to participant.)

< 1 month ago
1 to 6 months ago
6-12 months ago
More than 12 months ago
Don’t know
Declined to answer

5.19) Have you ever eaten a bat?
Yes
No
Don't know
Declined to answer

5.20) If you ever eaten a bat
5.20.1) How often do you eat bats? (Note to interviewer: Read all but last two options to participant.)
once per year or less
2-4 times per year
5 times or more per year
Don’t know
Declined to answer

5.20.2) When was the last time you ate a bat?
< 1 month ago
1 to 6 months ago
6-12 months ago
More than 12 months ago
Don’t know
Declined to answer

5.21) What kinds of bats do you most frequently observe or have had contact with? (Note to interviewer: Read all but last two options to participant.)

Fruit-eating bats
Insect-eating bats
Vampire bats
Multiple types
Other
Don’t know
Declined to answer

5.22) If the kind of bat is "Other"
5.22.1) Specify the other type of bat

5.23) Do you or your family do something to avoid bat bites in the house?
Nothing
Use mosquito net
Prevent entry of bat in the house
Increase number of cats
Increase the number of cattle/pigs to be bitten
Destroy bat refuges/kill bats
Pray
Declined to answer
Other

5.24) If answered OTHER as the type of action taken to avoid bites in the house
5.24.1) Specify what you and your family does to avoid bat bites in the house

5.25) How much do you know about rabies? (Note to interviewer: Read all but last two options to participant.)
Little to none
Basic
Extensive
Declined to answer

5.26) How dangerous is rabies?
Very Severe
Mild or moderate
Don't know
Declined to answer

5.27) How do people get infected with rabies?
Animal bite
Animal scratch or lick
Touching an animal
Eating an animal
Other
Don't know
Declined to answer

5.28) If the way people are infected with rabies is "Other"
5.28.1) Specify the way people are infected by rabies

5.29) What animals can be infected with rabies? (check all that apply)
Bats
Dogs
Cats
Horses
Livestock

Wild mammals (not bats)
Other
Don't know
Declined to answer

5.30) If the animals are potentially infected with rabies are "Other"
5.30.1) Specify which other animals could be infected with rabies

5.31) What would you do if you were bitten or scratched by a bat?
Nothing
Wash wound with soap and water
Call a doctor for advice
Call or visit a traditional healer
Seek medical care at a hospital, clinic or health post
Seek rabies PEP (rabies vaccine)
Have bat tested for rabies (or other diseases)
Other
Don’t know
Declined to answer

5.32) If the action that you would take is Other
5.32.1) Specify the other action that would be taken

5.33) Do you think there is any time of the year in which bats attack more animals or people?
No, it is the same all year round
Yes, rainy season (April-October)
Yes, dry season (November-April)
Don't know
Declined to answer

5.34) If someone has been bitten by an animal potentially infected with rabies what should that person do? (Check all that apply)
Nothing
Wash wound with soap and water
Call a doctor for advice
Call or visit a traditional healer
Seek medical care at a hospital or clinic
Seek rabies PEP (rabies vaccines)
Check animal’s vaccination history
Observe animal for a period of time to see if it becomes rabid
Have animal tested for rabies
Kill animal
Other
Don't know

Declined to answer

5.35) If the action is "Other"

5.35.1) Specify the other action that should be done if someone has been bitten by an animal that might be infected by rabies

5.36) Have you ever been vaccinated against rabies?

Yes
No
Don't know
Declined to answer

5.37) If you have ever been vaccinated against rabies

5.37.1) What was the reason you were vaccinated against rabies?

Post-exposure prophylaxis
Pre-exposure prophylaxis
Have received PreP and PEP
Don’t know
Declined to answer

5.37.2) If you have received rabies vaccination after being bitten or scratched by an animal bite, what animal or animals were responsible for the incident? (check all that apply)

Bats
Dogs
Cats
Horses
Livestock
Wild mammals (not bats)
Others
Don't know
Declined to answer
Did not receive PEP

5.37.3) If received a vaccination after being bitten by an OTHER animal

5.37.3.1) Specify the other animal that bit you

5.38) Are you aware if there are any other diseases that humans can get from bats?

Yes
No
Don't know
Declined to answer

5.39) Have you or anyone you know ever experienced an illness that you believe may have been caused by bats or being in a bat cave?

Yes
No
Don't know
Declined to answer

5.40) If you or anyone you know ever experienced an illness that you believe may have been caused by bats or being in a bat cave

5.40.1) What were the symptoms?

Skin rash/discoloration/ infection
Unusual bleeding (e.g. from nose/mouth)
Fever
Cough
Sneezing
Runny nose
Chest congestion
Muscle pain
Difficulty breathing
Headache
Convulsions
Altered mental state (dementia)
Unconsciousness/coma
Muscle weakness/paralysis
Vomiting or diarrhea or stomach cramps
Miscarriage/stillbirth
Death
Multiple persons
Other
Don't know
Declined to answer

5.40.2) If the symptoms is "Other"

5.40.2.1) Specify the other symptom

5.40.2.2) Are you or the person you know that presented symptoms caused by a bat recovered?

Yes
No
Don’t know
Decline to answer

5.41) We would like to take a sample of your blood. Will you allow us to take a sample?

Yes
No

5.41.2 Was blood sampled obtained?

Yes
No
5.41.3 IF blood sample was not obtained, why not?
Did not consent for blood
Was not able to get blood
Other:_______________
5.41.4) Will you allow us to return in 6-8 weeks to ask you some more questions? YES/ NO

6) Please ask again if there is anyone else living here that has been bitten, scratched or has eaten or touched any bats. If so then fill additional section C for each additional exposed person. (Follow same process for consent/assent and blood sampling than other participants.

Those are all the questions I have for you. Thank you very much for your time and cooperation. We or personnel of the MoH may need to contact you again if the survey is found to be incomplete. Results of this study will be reported to MoH representatives in your area.

Technical Appendix 2. Questionnaire used in a follow-up survey of bat exposures — Idanre, Nigeria, 2013

D	D	M	M	Y	Y	Y	Y

Date of Follow-up: Household ID Number:
(autofill)

Interviewer Name: First Name, First Family name, Second Family name

1. Municipality: autofill
2. Community: autofill
3. GPS Coordinates: autofill

Section A

[Section A. Administer to the person originally consented to the main responder of the study. If not available, ask if another adult (18 years and older) is available]

Hello. My name is ____________________ and I am working with the <insert appropriate agency affiliation>.

Mr./Mrs. (*name of person originally consented to the study*) participated in a survey in Feb/March of this year; is (*he/she*) in the house and available to participate in a follow-up survey at this time?

If available, interviewer to confirm that consent was obtained for participation in the Feb/March survey (Yes, No)<br>If not available, ask if another adult who participate in the original study is available to answer follow-up questions.

Last (Feb/March) (*you* or *name of person originally consented to the study)* agreed to participate in a survey to improve our understanding of the knowledge, attitudes, and practices of people in Idanre local government of Ondo State, who come in close contact with bats or places where bats live, like caves. We are here today to ask for a few more minutes of your time, around 20 minutes, to follow-up on your responses about any animals you've kept as pets or livestock since the festival, exposures to bats since the festival, and about your health since the bat festival. Your answers to the questions are completely voluntary and will be kept confidential. Do you have

time now? (If NO, "Thank you for your time." Ask if there is another time that would be more convenient)
Just like for the first survey, you do not have to be in this follow-up survey. It is up to you. You do not have to answer any question or give blood if you do not want to.

Do you want to be in the follow-up part of the Nigeria Bats study? (Yes, No)

Name:____
Signature:____________________________

Date:____ Right Thumbprint (if not able to read/write): ____________

Please think carefully about each question, and answer as well as you can. You can choose not to answer any of the questions.

History of Animal Illness Since the Bat Festival:

1. A) At the time of the bat festival, did you have any animals as pets or livestock? (Yes, No, Don't know, Declined to answer)

   If no, go to **Section B** on page 6
   If yes, ask the following questions:

   B) Have any of the animals died since the festival? (Yes, No, Don't know, Declined to answer)
   C) Have any of the animals been sick since the festival? (Yes, No, Don't know, Declined to answer)
   D) During or since the bat festival, did any of your animals come in contact with bats – either by biting, scratching, or touching (Yes, No, Don't know, Declined to answer)
   E) If yes, please indicate which sort of the animals have been in contact with bats during or since the bat festival (Select all that apply?)

Goats	Dogs
Sheep	Cats
Cows	Chicken
Pigs	Other (Specify "other" type of animal)
Horses	

F) Now I/we are going to ask you more about the animals you had at the time of the festival and any sickness or death they've had since the festival.

Household ID# | | | | | | | | |

Follow-up Form
(Convalescent Blood Draw Visit)

Complete for each species	Questions about animal sickness	Clinical signs? (tick all that apply)	Questions about animal death
**I. Goats:**<br>How many total? (number, N/A, don't know, declined, N/A)	a) How many got sick?<br>☐ 0 ☐ 1<br>☐ 2 ☐ 3+<br>☐ D/K ☐ Declined<br>b) Is/are the animal recovered from the symptoms? (yes, no, unsure, declined)	c) What were their signs? (tick all that apply)<br>☐ Not moving much/hiding ☐ Problems walking<br>☐ Not eating well ☐ Vomiting ☐ Diarrhea<br>☐ Foaming at mouth/salivation<br>☐ Bellowing/crying ☐ Trembling or twitching<br>☐ Behavior change – more quiet/more aggressive ☐ Coughing ☐ Sneezing ☐ Runny nose<br>☐ Problems breathing ☐ Convulsions<br>☐ Still birth ☐ Suddenly died<br>☐ Other (specify):	d) How many died?<br>☐ 0 ☐ 1<br>☐ 2 ☐ 3+<br>☐ D/K ☐ Declined<br>e) Was/were the animal(s) slaughtered and eaten or sold for food? (yes, no, some, unsure, declined)<br>f) Was/were any of the animals sick before they were slaughtered or sold? (yes, no, some, unsure, declined)
**II. Sheep**<br>How many total? (number, N/A, don't know, declined, N/A)	a) How many got sick?<br>☐ 0 ☐ 1<br>☐ 2 ☐ 3+<br>☐ D/K ☐ Declined<br>b) Is/are the animal recovered from the symptoms? (yes, no, unsure, declined)	c) What were their signs? (tick all that apply)<br>☐ Not moving much/hiding ☐ Problems walking<br>☐ Not eating well ☐ Vomiting ☐ Diarrhea<br>☐ Foaming at mouth/salivation<br>☐ Bellowing/crying ☐ Trembling or twitching<br>☐ Behavior change – more quiet/more aggressive ☐ Coughing ☐ Sneezing ☐ Runny nose<br>☐ Problems breathing ☐ Convulsions<br>☐ Still birth ☐ Suddenly died<br>☐ Other (specify):	d) How many died?<br>☐ 0 ☐ 1<br>☐ 2 ☐ 3+<br>☐ D/K ☐ Declined<br>e) Was/were the animal(s) slaughtered and eaten or sold for food? (yes, no, some, unsure, declined)<br>f) Was/were any of the animals sick before they were slaughtered or sold? (yes, no, some, unsure, declined)

**III. Cows**<br>How many total? (number, N/A, don't know, declined, N/A)	a) How many got sick?<br>☐ 0 ☐ 1<br>☐ 2 ☐ 3+<br>☐ D/K ☐ Declined<br>b) Is/are the animal recovered from the symptoms? (yes, no, unsure, declined)	c) What were their signs? (tick all that apply)<br>☐ Not moving much/hiding ☐ Problems walking<br>☐ Not eating well ☐ Vomiting ☐ Diarrhea<br>☐ Foaming at mouth/salivation<br>☐ Bellowing/crying ☐ Trembling or twitching<br>☐ Behavior change – more quiet/more aggressive ☐ Coughing ☐ Sneezing ☐ Runny nose<br>☐ Problems breathing ☐ Convulsions<br>☐ Still birth ☐ Suddenly died<br>☐ Other (specify):	d) How many died?<br>☐ 0 ☐ 1<br>☐ 2 ☐ 3+<br>☐ D/K ☐ Declined<br>e) Was/were the animal(s) slaughtered and eaten or sold for food? (yes, no, some, unsure, declined)<br>f) Was/were any of the animals sick before they were slaughtered or sold? (yes, no, some, unsure, declined)
**IV. Pigs**<br>How many total? (number, N/A, don't know, declined, N/A)	a) How many got sick?<br>☐ 0 ☐ 1<br>☐ 2 ☐ 3+<br>☐ D/K ☐ Declined<br>b) Is/are the animal recovered from the symptoms? (yes, no, unsure, declined)	c) What were their signs? (tick all that apply)<br>☐ Not moving much/hiding ☐ Problems walking<br>☐ Not eating well ☐ Vomiting ☐ Diarrhea<br>☐ Foaming at mouth/salivation<br>☐ Bellowing/crying ☐ Trembling or twitching<br>☐ Behavior change – more quiet/more aggressive ☐ Coughing ☐ Sneezing ☐ Runny nose<br>☐ Problems breathing ☐ Convulsions<br>☐ Still birth ☐ Suddenly died<br>☐ Other (specify):	d) How many died?<br>☐ 0 ☐ 1<br>☐ 2 ☐ 3+<br>☐ D/K ☐ Declined<br>e) Was/were the animal(s) slaughtered and eaten or sold for food? (yes, no, some, unsure, declined)<br>f) Was/were any of the animals sick before they were slaughtered or sold? (yes, no, some, unsure, declined)
**V. Horses**<br>How many total? (number, N/A,	a) How many got sick?<br>☐ 0 ☐ 1<br>☐ 2 ☐ 3+	c) What were their signs? (tick all that apply)<br>☐ Not moving much/hiding ☐ Problems walking	d) How many died?<br>☐ 0 ☐ 1<br>☐ 2 ☐ 3+

don't know, declined, N/A)	☐ D/K ☐ Declined<br><br>b) Is/are the animal recovered from the symptoms? (yes, no, unsure, declined)	☐ Not eating well ☐ Vomiting ☐ Diarrhea<br>☐ Foaming at mouth/salivation<br>☐ Bellowing/crying ☐ Trembling or twitching<br>☐ Behavior change – more quiet/more aggressive ☐ Coughing ☐ Sneezing ☐ Runny nose<br>☐ Problems breathing ☐ Convulsions<br>☐ Still birth ☐ Suddenly died<br>☐ Other (specify):	☐ D/K ☐ Declined<br><br>e) Was/were the animal(s) slaughtered and eaten or sold for food? (yes, no, some, unsure, declined)<br><br>f) Was/were any of the animals sick before they were slaughtered or sold? (yes, no, some, unsure, declined)
**VI. Dogs**<br><br>How many total? (number, N/A, don't know, declined, N/A)	a) How many got sick?<br><br>☐ 0 ☐ 1<br>☐ 2 ☐ 3+<br>☐ D/K ☐ Declined<br><br>b) Is/are the animal recovered from the symptoms? (yes, no, unsure, declined)	c) What were their signs? (tick all that apply)<br><br>☐ Not moving much/hiding ☐ Problems walking<br>☐ Not eating well ☐ Vomiting ☐ Diarrhea<br>☐ Foaming at mouth/salivation<br>☐ Bellowing/crying ☐ Trembling or twitching<br>☐ Behavior change – more quiet/more aggressive ☐ Coughing ☐ Sneezing ☐ Runny nose<br>☐ Problems breathing ☐ Convulsions<br>☐ Still birth ☐ Suddenly died<br>☐ Other (specify):	d) How many died?<br><br>☐ 0 ☐ 1<br>☐ 2 ☐ 3+<br>☐ D/K ☐ Declined<br><br>e) Was/were the animal(s) slaughtered and eaten or sold for food? (yes, no, some, unsure, declined)<br><br>f) Was/were any of the animals sick before they were slaughtered or sold? (yes, no, some, unsure, declined)

**VII. Cats**	a) How many got sick?<br><br>☐ 0 ☐ 1<br>☐ 2 ☐ 3+	c) What were their signs? (tick all that apply)<br><br>☐ Not moving much/hiding ☐ Problems walking	d) How many died?<br><br>☐ 0 ☐ 1<br>☐ 2 ☐ 3+

How many total? (number, N/A, don't know, declined, N/A)	☐ D/K ☐ Declined<br><br>b) Is/are the animal recovered from the symptoms? (yes, no, unsure, declined)	☐ Not eating well ☐ Vomiting ☐ Diarrhea<br>☐ Foaming at mouth/salivation<br>☐ Bellowing/crying ☐ Trembling or twitching<br>☐ Behavior change – more quiet/more aggressive ☐ Coughing ☐ Sneezing ☐ Runny nose<br>☐ Problems breathing ☐ Convulsions<br>☐ Still birth ☐ Suddenly died<br>☐ Other (specify):	☐ D/K ☐ Declined<br><br>e) Was/were the animal(s) slaughtered and eaten or sold for food? (yes, no, some, unsure, declined)<br><br>f) Was/were any of the animals sick before they were slaughtered or sold? (yes, no, some, unsure, declined
**VIII. Other** (specify)<br><br>How many total? (number, N/A, don't know, declined, N/A)	a) How many got sick?<br><br>☐ 0 ☐ 1<br>☐ 2 ☐ 3+<br>☐ D/K ☐ Declined<br><br>b) Is/are the animal recovered from the symptoms? (yes, no, unsure, declined)	c) What were their signs? (tick all that apply)<br><br>☐ Not moving much/hiding ☐ Problems walking<br>☐ Not eating well ☐ Vomiting ☐ Diarrhea<br>☐ Foaming at mouth/salivation<br>☐ Bellowing/crying ☐ Trembling or twitching<br>☐ Behavior change – more quiet/more aggressive ☐ Coughing ☐ Sneezing ☐ Runny nose<br>☐ Problems breathing ☐ Convulsions<br>☐ Still birth ☐ Suddenly died<br>☐ Other (specify):	d) How many died?<br><br>☐ 0 ☐ 1<br>☐ 2 ☐ 3+<br>☐ D/K ☐ Declined<br><br>e) Was/were the animal(s) slaughtered and eaten or sold for food? (yes, no, some, unsure, declined)<br><br>f) Was/were any of the animals sick before they were slaughtered or sold? (yes, no, some, unsure, declined
**IX. Other** (specify)<br><br>How many total? (number, N/A, don't know, declined, N/A)	a) How many got sick?<br><br>☐ 0 ☐ 1<br>☐ 2 ☐ 3+<br>☐ D/K ☐ Declined<br><br>b) Is/are the animal recovered from the	c) What were their signs? (tick all that apply)<br><br>☐ Not moving much/hiding ☐ Problems walking<br>☐ Not eating well ☐ Vomiting ☐ Diarrhea<br>☐ Foaming at mouth/salivation	d) How many died?<br><br>☐ 0 ☐ 1<br>☐ 2 ☐ 3+<br>☐ D/K ☐ Declined<br><br>e) Was/were the animal(s) slaughtered and eaten or sold for

	symptoms? (yes, no, unsure, declined)	☐ Bellowing/crying ☐ Trembling or twitching<br>☐ Behavior change – more quiet/more aggressive ☐ Coughing ☐ Sneezing ☐ Runny nose<br>☐ Problems breathing ☐ Convulsions<br>☐ Still birth ☐ Suddenly died<br>☐ Other (specify):	food? (yes, no, some, unsure, declined)<br>f) Was/were any of the animals sick before they were slaughtered or sold? (yes, no, some, unsure, declined

Section B: Household Respondent Information:

Now we would like to ask you and the members of your household who participated in the last study, if they would like to like to answer questions about their exposure to bats and health status since the festival. Answers to the questions are completely voluntary and will be kept confidential.

2. Patient ID: (autofill)
3. Sample ID: (autofill)

D	D	M	M	Y	Y	Y	Y

4. Date of Follow-up:

Follow-up Form
(Convalescent Blood Draw Visit)

Interviewer to confirm the following information:

5. Name: First name, First Family name, Second Family name (autofill)
6. Respondent Age (autofill: age in years)
7. Confirm patient gender (autofill: male/female)
8. Contact / Mobile Number:

9. A) Respondent Status: Alive/Deceased
   B) If deceased, specify source of information:

Bat Exposure During and Since Bat Festival:

10. A) Did you participate in the last bat festival (specify dates)? (Yes (date/s), No, don’t know, declined)
    If Yes, ask the following questions:
    B) What dates did you participate? (Date/s, don’t know, declined)
    C) What role(s) did you play during the bat festival? (tick all that concerns)

Bat hunting	Dancer, singer	Decline to answer
Selling of bats	Spiritual activities	Other (specify) ____________
Preparation of bats for food/consumption	Standby watcher	
	Don’t know	

5.

11. A) Did you go inside of a bat cave or bat refuge during or after the festival (trees, abandoned house, bridge, etc.)?
    (Yes, No, Don't know, Declined to answer)
    If yes, ask the following questions:
    B) How many times did you enter a bat cave or bat refuge during the festival? (*N*, Don’t’ know, declined to answer)
    C) How many times did you enter a bat cave or bat refuge since the festival? (*N*, Don’t’ know, declined to answer)
    D) When was the last time you entered a bat cave or refuge? (Note to interviewer: Read all but last two options to participant.)
        During the festival

Since after the festival: 1-4 weeks ago (in the past 4 weeks)
Since after the festival: 5-8 weeks ago (longer than 4 weeks ago)
Don't know
Declined to answer

12. A) During or since the bat festival, have you touched a live bat with your skin uncovered? (Yes, No, Don't know, Declined to answer)
B) If yes, when was the last time you touched a bat?
During the festival
Since after the festival: 1-4 weeks ago (in the past 4 weeks)
Since after the festival: 5-8 weeks ago
Don't know
Declined to answer

13. A) During or since the bat festival, were you scratched by a bat, to your knowledge? (Yes, No, Don't know, Declined to answer)
B) If yes, when was the last time you were scratched by a bat?
During the festival
Since after the festival: 1-4 weeks ago (in the past 4 weeks)
Since after the festival: 5-8 weeks ago
Don't know
Declined to answer

14. A) During or since the bat festival, were you bitten by a bat, to your knowledge? (Yes, No, Don't know, Declined to answer)
    B) If yes, when was the last time you were bitten by a bat?
    - During the festival
    - Since after the festival: 1-4 weeks ago (in the past 4 weeks)
    - Since after the festival: 5-8 weeks ago
    - Don't know
    - Declined to answer
15. A) During or since the bat festival, did you prepare bat as food? (Yes, No, Don't know, Declined to answer)
    B) If yes, when was the last time you prepared bat as food?
    - During the festival
    - Since after the festival: 1-4 weeks ago (in the past 4 weeks)
    - Since after the festival: 5-8 weeks ago
    - Don't know
    - Declined to answer
16. A) During or since the bat festival, did you eat bat? (Yes, No, Don't know, Declined to answer)
    B) If yes, when was the last time you ate bat?
    - During the festival
    - Since after the festival: 1-4 weeks ago (in the past 4 weeks)
    - Since after the festival: 5-8 weeks ago
    - Don't know
    - Declined to answer
17. What kinds of bats do you most frequently observe or have had contact with? (Note to interviewer: Read all but last two options to participant.)
    - Fruit-eating bats
    - Insect-eating bats
    - Vampire bats
    - Multiple types
    - Other (specify)
    - Don't know

Declined to answer

Respondent History of Illness Since Bat Festival:

18. A) Since the bat festival, have you felt sick at any time? Yes, No

B) If yes, did you go for help when you felt sick? (Y=1, N=2, Declined=99)

If yes: ask the following questions:

a) Where did you go? (nearby clinic, state hospital, private hospital/clinic, pharmacy/chemist, traditional healer, other: (specify))

b) What did the doctor/healer/chemist say was wrong? (list all, unsure=3, declined=99)

c) Did you stay at the hospital for treatment? (Y=1, N=2, declined=99)

d) If yes, how many days were you in the hospital?

e) Did the doctor/healer/chemist prescribe any medication?

i) If yes, what medication/s: (list all, unsure=3, declined=99)

19. A) Since the bat festival, have you taken any medications?

B) If yes, what medication/s: (list all, unsure=3, declined=99)

Follow-up Form
(Convalescent Blood Draw Visit)

20. Now I/we would like to ask you some questions about the symptoms you had when you were sick after the bat festival

ORY OF ILLNESS	Have you had "*name specific symptom*" (Yes = 1, No= 2, unsure= 3, declined =99)	How many days ago did it start? (if started today: code=00, NA=88, declined =99)	How many days did the symptom last? (if continuing until today, count current day as 1; NA= 88, declined=99)	Did you have this symptom before o during the bat fes (Yes=1, No=2, unsure=3, NA=88, declined=99)
:				
a:				
ing:				
ea:				
minal pain:				
nctivitis/red eye:				
n:				
lcers or cold sores:				
hroat:				
ulty breathing:				
pain:				
e aches:				
ain:				
ired/weak:				
che:				
______________________				
ng from gums or mouth				
ORY OF ILLNESS	Have you had "*name specific symptom*" (Yes = 1, No= 2, unsure= 3, declined =99)	How many days ago did it start? (if started today: code=00, NA=88, declined =99)	How many days did the symptom last? (if continuing until today, count current day as 1; NA= 88, declined=99)	Did you have this symptom before o during the bat fes (Yes=1, No=2, unsure=3, NA=88, declined=99)
spots in eyes (sclera) or on skin				

Follow-up Form
(Convalescent Blood Draw Visit)

eck:				
anced/difficulty walking:				
ulty swallowing:				
ulty speaking:				
ulty hearing:				
ulty seeing:				
sive fear/anxiety				
ed				
rs or convulsions:				
d or loss of consciousness:				
sis:				
symptom 1, y____________________				
symptom 2, y____________________				

21. A) Since the last time we talked to you around 2 months ago, have you received rabies vaccination? Yes, No

B) If yes, when were you given your last rabies vaccination?”

Those are all the questions I have for you. Thank you very much for your time and cooperation. We or personnel of the state Ministry of Health may need to contact you again if the survey is found to be incomplete. Results of this study will be reported to Ministry of Health representatives in your area.

Questionnaire used in a follow-up survey of bat exposures — Idanre, Nigeria, 2013

Date of Follow-up:

D	D	M	M	Y	Y	Y	Y

Household ID Number: (autofill)

Interviewer Name: First Name, First Family name, Second Family name

1. Municipality: autofill
2. Community: autofill
3. GPS Coordinates: autofill

Section A

[Section A. Administer to the person originally consented to the main responder of the study. If not available, ask if another adult (18 years and older) is available]

Hello. My name is ____________________ and I am working with the <insert appropriate agency affiliation>.

Mr./Mrs. (*name of person originally consented to the study*) participated in a survey in Feb/March of this year; is (*he/she*) in the house and available to participate in a follow-up survey at this time?

If available, interviewer to confirm that consent was obtained for participation in the Feb/March survey (Yes, No)

If not available, ask if another adult who participate in the original study is available to answer follow-up questions.

Last (Feb/March) (*you* or *name of person originally consented to the study)* agreed to participate in a survey to improve our understanding of the knowledge, attitudes, and practices of people in Idanre local government of Ondo State, who come in close contact with bats or places where bats live, like caves. We are here today to ask for a few more minutes of your time, around 20 minutes, to follow-up on your responses about any animals you've kept as pets or livestock since the festival, exposures to bats since the festival, and about your health since the bat festival. Your answers to the questions are completely voluntary and will be kept confidential. Do you have time now? (If NO, "Thank you for your time." Ask if there is another time that would be more convenient)

Just like for the first survey, you do not have to be in this follow-up survey. It is up to you. You do not have to answer any question or give blood if you do not want to.

Do you want to be in the follow-up part of the Nigeria Bats study? (Yes, No)

Name:____ Signature:____________________________

Date:____ Right Thumbprint (if not able to read/write): ____________

Please think carefully about each question, and answer as well as you can. You can choose not to answer any of the questions.

History of Animal Illness Since the Bat Festival:

1. A) At the time of the bat festival, did you have any animals as pets or livestock? (Yes, No, Don't know, Declined to answer)

   If no, go to **Section B** on page 6

Household ID# | | | | | | | |

If yes, ask the following questions:

B) Have any of the animals died since the festival? (Yes, No, Don't know, Declined to answer)

C) Have any of the animals been sick since the festival? (Yes, No, Don't know, Declined to answer)

D) During or since the bat festival, did any of your animals come in contact with bats – either by biting, scratching, or touching (Yes, No, Don't know, Declined to answer)

E) If yes, please indicate which sort of the animals have been in contact with bats during or since the bat festival (Select all that apply?)

Goats	Dogs
Sheep	Cats
Cows	Chicken
Pigs	Other (Specify "other" type of animal)
Horses	

F) Now I/we are going to ask you more about the animals you had at the time of the festival and any sickness or death they've had since the festival.

Household ID# | | | | | | | | |

Follow-up Form
(Convalescent Blood Draw Visit)

Complete for each species	Questions about animal sickness	Clinical signs? (tick all that apply)	Questions about animal death
**I. Goats:**<br>How many total? (number, N/A, don't know, declined, N/A)	a) How many got sick?<br>☐ 0 ☐ 1<br>☐ 2 ☐ 3+<br>☐ D/K ☐ Declined<br>b) Is/are the animal recovered from the symptoms? (yes, no, unsure, declined)	c) What were their signs? (tick all that apply)<br>☐ Not moving much/hiding ☐ Problems walking<br>☐ Not eating well ☐ Vomiting ☐ Diarrhea<br>☐ Foaming at mouth/salivation<br>☐ Bellowing/crying ☐ Trembling or twitching<br>☐ Behavior change – more quiet/more aggressive<br>☐ Coughing ☐ Sneezing ☐ Runny nose<br>☐ Problems breathing ☐ Convulsions<br>☐ Still birth ☐ Suddenly died<br>☐ Other (specify):	d) How many died?<br>☐ 0 ☐ 1<br>☐ 2 ☐ 3+<br>☐ D/K ☐ Declined<br>e) Was/were the animal(s) slaughtered and eaten or sold for food? (yes, no, some, unsure, declined)<br>f) Was/were any of the animals sick before they were slaughtered or sold? (yes, no, some, unsure, declined)
**II. Sheep**<br>How many total? (number, N/A, don't know, declined, N/A)	a) How many got sick?<br>☐ 0 ☐ 1<br>☐ 2 ☐ 3+<br>☐ D/K ☐ Declined<br>b) Is/are the animal recovered from the symptoms? (yes, no, unsure, declined)	c) What were their signs? (tick all that apply)<br>☐ Not moving much/hiding ☐ Problems walking<br>☐ Not eating well ☐ Vomiting ☐ Diarrhea<br>☐ Foaming at mouth/salivation<br>☐ Bellowing/crying ☐ Trembling or twitching<br>☐ Behavior change – more quiet/more aggressive<br>☐ Coughing ☐ Sneezing ☐ Runny nose<br>☐ Problems breathing ☐ Convulsions<br>☐ Still birth ☐ Suddenly died<br>☐ Other (specify):	d) How many died?<br>☐ 0 ☐ 1<br>☐ 2 ☐ 3+<br>☐ D/K ☐ Declined<br>e) Was/were the animal(s) slaughtered and eaten or sold for food? (yes, no, some, unsure, declined)<br>f) Was/were any of the animals sick before they were slaughtered or sold? (yes, no, some, unsure, declined)

Household ID# ☐☐☐☐☐☐☐☐

Follow-up Form
(Convalescent Blood Draw Visit)

**III. Cows**<br>How many total? (number, N/A, don't know, declined, N/A)	a) How many got sick?<br>☐ 0 ☐ 1<br>☐ 2 ☐ 3+<br>☐ D/K ☐ Declined<br>b) Is/are the animal recovered from the symptoms? (yes, no, unsure, declined)	c) What were their signs? (tick all that apply)<br>☐ Not moving much/hiding ☐ Problems walking<br>☐ Not eating well ☐ Vomiting ☐ Diarrhea<br>☐ Foaming at mouth/salivation<br>☐ Bellowing/crying ☐ Trembling or twitching<br>☐ Behavior change – more quiet/more aggressive<br>☐ Coughing ☐ Sneezing ☐ Runny nose<br>☐ Problems breathing ☐ Convulsions<br>☐ Still birth ☐ Suddenly died<br>☐ Other (specify):	d) How many died?<br>☐ 0 ☐ 1<br>☐ 2 ☐ 3+<br>☐ D/K ☐ Declined<br>e) Was/were the animal(s) slaughtered and eaten or sold for food? (yes, no, some, unsure, declined)<br>f) Was/were any of the animals sick before they were slaughtered or sold? (yes, no, some, unsure, declined)
**IV. Pigs**<br>How many total? (number, N/A, don't know, declined, N/A)	a) How many got sick?<br>☐ 0 ☐ 1<br>☐ 2 ☐ 3+<br>☐ D/K ☐ Declined<br>b) Is/are the animal recovered from the symptoms? (yes, no, unsure, declined)	c) What were their signs? (tick all that apply)<br>☐ Not moving much/hiding ☐ Problems walking<br>☐ Not eating well ☐ Vomiting ☐ Diarrhea<br>☐ Foaming at mouth/salivation<br>☐ Bellowing/crying ☐ Trembling or twitching<br>☐ Behavior change – more quiet/more aggressive<br>☐ Coughing ☐ Sneezing ☐ Runny nose<br>☐ Problems breathing ☐ Convulsions<br>☐ Still birth ☐ Suddenly died<br>☐ Other (specify):	d) How many died?<br>☐ 0 ☐ 1<br>☐ 2 ☐ 3+<br>☐ D/K ☐ Declined<br>e) Was/were the animal(s) slaughtered and eaten or sold for food? (yes, no, some, unsure, declined)<br>f) Was/were any of the animals sick before they were slaughtered or sold? (yes, no, some, unsure, declined)
**V. Horses**<br>How many total? (number, N/A,	a) How many got sick?<br>☐ 0 ☐ 1	c) What were their signs? (tick all that apply)<br>☐ Not moving much/hiding ☐ Problems walking	d) How many died?<br>☐ 0 ☐ 1

don't know, declined, N/A)	☐ 2 ☐ 3+<br>☐ D/K ☐ Declined<br><br>b) Is/are the animal recovered from the symptoms? (yes, no, unsure, declined)	☐ Not eating well ☐ Vomiting ☐ Diarrhea<br>☐ Foaming at mouth/salivation<br>☐ Bellowing/crying ☐ Trembling or twitching<br>☐ Behavior change – more quiet/more aggressive<br>☐ Coughing ☐ Sneezing ☐ Runny nose<br>☐ Problems breathing ☐ Convulsions<br>☐ Still birth ☐ Suddenly died<br>☐ Other (specify):	☐ 2 ☐ 3+<br>☐ D/K ☐ Declined<br>e) Was/were the animal(s) slaughtered and eaten or sold for food? (yes, no, some, unsure, declined)<br>f) Was/were any of the animals sick before they were slaughtered or sold? (yes, no, some, unsure, declined)
**VI. Dogs**<br>How many total? (number, N/A, don't know, declined, N/A)	a) How many got sick?<br>☐ 0 ☐ 1<br>☐ 2 ☐ 3+<br>☐ D/K ☐ Declined<br><br>b) Is/are the animal recovered from the symptoms? (yes, no, unsure, declined)	c) What were their signs? (tick all that apply)<br>☐ Not moving much/hiding ☐ Problems walking<br>☐ Not eating well ☐ Vomiting ☐ Diarrhea<br>☐ Foaming at mouth/salivation<br>☐ Bellowing/crying ☐ Trembling or twitching<br>☐ Behavior change – more quiet/more aggressive<br>☐ Coughing ☐ Sneezing ☐ Runny nose<br>☐ Problems breathing ☐ Convulsions<br>☐ Still birth ☐ Suddenly died<br>☐ Other (specify):	d) How many died?<br>☐ 0 ☐ 1<br>☐ 2 ☐ 3+<br>☐ D/K ☐ Declined<br>e) Was/were the animal(s) slaughtered and eaten or sold for food? (yes, no, some, unsure, declined)<br>f) Was/were any of the animals sick before they were slaughtered or sold? (yes, no, some, unsure, declined)

Follow-up Form
(Convalescent Blood Draw Visit)

**VII. Cats**<br>How many total? (number, N/A, don't know, declined, N/A)	a) How many got sick?<br>☐ 0 ☐ 1<br>☐ 2 ☐ 3+<br>☐ D/K ☐ Declined<br>b) Is/are the animal recovered from the symptoms? (yes, no, unsure, declined)	c) What were their signs? (tick all that apply)<br>☐ Not moving much/hiding ☐ Problems walking<br>☐ Not eating well ☐ Vomiting ☐ Diarrhea<br>☐ Foaming at mouth/salivation<br>☐ Bellowing/crying ☐ Trembling or twitching<br>☐ Behavior change – more quiet/more aggressive<br>☐ Coughing ☐ Sneezing ☐ Runny nose<br>☐ Problems breathing ☐ Convulsions<br>☐ Still birth ☐ Suddenly died<br>☐ Other (specify):	d) How many died?<br>☐ 0 ☐ 1<br>☐ 2 ☐ 3+<br>☐ D/K ☐ Declined<br>e) Was/were the animal(s) slaughtered and eaten or sold for food? (yes, no, some, unsure, declined)<br>f) Was/were any of the animals sick before they were slaughtered or sold? (yes, no, some, unsure, declined
**VIII. Other** (specify)<br>How many total? (number, N/A, don't know, declined, N/A)	a) How many got sick?<br>☐ 0 ☐ 1<br>☐ 2 ☐ 3+<br>☐ D/K ☐ Declined<br>b) Is/are the animal recovered from the symptoms? (yes, no, unsure, declined)	c) What were their signs? (tick all that apply)<br>☐ Not moving much/hiding ☐ Problems walking<br>☐ Not eating well ☐ Vomiting ☐ Diarrhea<br>☐ Foaming at mouth/salivation<br>☐ Bellowing/crying ☐ Trembling or twitching<br>☐ Behavior change – more quiet/more aggressive<br>☐ Coughing ☐ Sneezing ☐ Runny nose<br>☐ Problems breathing ☐ Convulsions<br>☐ Still birth ☐ Suddenly died<br>☐ Other (specify):	d) How many died?<br>☐ 0 ☐ 1<br>☐ 2 ☐ 3+<br>☐ D/K ☐ Declined<br>e) Was/were the animal(s) slaughtered and eaten or sold for food? (yes, no, some, unsure, declined)<br>f) Was/were any of the animals sick before they were slaughtered or sold? (yes, no, some, unsure, declined
**IX. Other** (specify)	a) How many got sick?	c) What were their signs? (tick all that apply)	d) How many died?

How many total? (number, N/A, don't know, declined, N/A)	☐ 0 ☐ 1<br>☐ 2 ☐ 3+<br>☐ D/K ☐ Declined<br><br>b) Is/are the animal recovered from the symptoms? (yes, no, unsure, declined)	☐ Not moving much/hiding ☐ Problems walking<br>☐ Not eating well ☐ Vomiting ☐ Diarrhea<br>☐ Foaming at mouth/salivation<br>☐ Bellowing/crying ☐ Trembling or twitching<br>☐ Behavior change – more quiet/more aggressive<br>☐ Coughing ☐ Sneezing ☐ Runny nose<br>☐ Problems breathing ☐ Convulsions<br>☐ Still birth ☐ Suddenly died<br>☐ Other (specify):	☐ 0 ☐ 1<br>☐ 2 ☐ 3+<br>☐ D/K ☐ Declined<br>e) Was/were the animal(s) slaughtered and eaten or sold for food? (yes, no, some, unsure, declined)<br>f) Was/were any of the animals sick before they were slaughtered or sold? (yes, no, some, unsure, declined

Section B: Household Respondent Information:

Now we would like to ask you and the members of your household who participated in the last study, if they would like to like to answer questions about their exposure to bats and health status since the festival. Answers to the questions are completely voluntary and will be kept confidential.

2. Patient ID: (autofill)

3. Sample ID: (autofill)

4. Date of Follow-up:

D	D	M	M	Y	Y	Y	Y

Household ID#

Follow-up Form
(Convalescent Blood Draw Visit)

Interviewer to confirm the following information:

5. Name: First name, First Family name, Second Family name (autofill)
6. Respondent Age (autofill: age in years)
7. Confirm patient gender (autofill: male/female)
8. Contact / Mobile Number:

9. A) Respondent Status: Alive/Deceased
   B) If deceased, specify source of information:

Bat Exposure During and Since Bat Festival:

10. A) Did you participate in the last bat festival (specify dates)? (Yes (date/s), No, don't know, declined)
    If Yes, ask the following questions:

    B) What dates did you participate? (Date/s, don't know, declined)

    C) What role(s) did you play during the bat festival? (tick all that concerns)

Bat hunting	Dancer, singer	Don't know
Selling of bats	Spiritual activities	Decline to answer
Preparation of bats for food/consumption	Standby watcher	Other (specify) ____________

11. A) Did you go inside of a bat cave or bat refuge during or after the festival (trees, abandoned house, bridge, etc.)?
    (Yes, No, Don't know, Declined to answer)

    If yes, ask the following questions:

B) How many times did you enter a bat cave or bat refuge during the festival? (*N*, Don't' know, declined to answer)

C) How many times did you enter a bat cave or bat refuge since the festival? (*N*, Don't' know, declined to answer)

D) When was the last time you entered a bat cave or refuge? (Note to interviewer: Read all but last two options to participant.)

During the festival

Since after the festival: 1-4 weeks ago (in the past 4 weeks)

Since after the festival: 5-8 weeks ago (longer than 4 weeks ago)

Don't know

Declined to answer

12. A) During or since the bat festival, have you touched a live bat with your skin uncovered? (Yes, No, Don't know, Declined to answer)
B) If yes, when was the last time you touched a bat?

During the festival

Since after the festival: 1-4 weeks ago (in the past 4 weeks)

Since after the festival: 5-8 weeks ago

Don't know

Declined to answer

13. A) During or since the bat festival, were you scratched by a bat, to your knowledge? (Yes, No, Don't know, Declined to answer)
B) If yes, when was the last time you were scratched by a bat?

During the festival

Since after the festival: 1-4 weeks ago (in the past 4 weeks)

Since after the festival: 5-8 weeks ago

Don’t know

Declined to answer

14. A) During or since the bat festival, were you bitten by a bat, to your knowledge? (Yes, No, Don't know, Declined to answer)
    B) If yes, when was the last time you were bitten by a bat?

    During the festival

    Since after the festival: 1-4 weeks ago (in the past 4 weeks)

    Since after the festival: 5-8 weeks ago

    Don’t know

    Declined to answer

15. A) During or since the bat festival, did you prepare bat as food? (Yes, No, Don't know, Declined to answer)
    B) If yes, when was the last time you prepared bat as food?

    During the festival

    Since after the festival: 1-4 weeks ago (in the past 4 weeks)

    Since after the festival: 5-8 weeks ago

    Don’t know

    Declined to answer

16. A) During or since the bat festival, did you eat bat? (Yes, No, Don't know, Declined to answer)
    B) If yes, when was the last time you ate bat?

    During the festival

    Since after the festival: 1-4 weeks ago (in the past 4 weeks)

    Since after the festival: 5-8 weeks ago

    Don’t know

Declined to answer

17. What kinds of bats do you most frequently observe or have had contact with? (Note to interviewer: Read all but last two options to participant.)

Fruit-eating bats

Insect-eating bats

Vampire bats

Multiple types

Other (specify)

Don’t know

Declined to answer

Follow-up Form
(Convalescent Blood Draw Visit)

Respondent History of Illness Since Bat Festival:

18. A) Since the bat festival, have you felt sick at any time? Yes, No
    B) If yes, did you go for help when you felt sick? (Y=1, N=2, Declined=99)

    If yes: ask the following questions:

    a) Where did you go? (nearby clinic, state hospital, private hospital/clinic, pharmacy/chemist, traditional healer, other: (specify))

    b) What did the doctor/healer/chemist say was wrong? (list all, unsure=3, declined=99)

    c) Did you stay at the hospital for treatment? (Y=1, N=2, declined=99)

    d) If yes, how many days were you in the hospital?

    e) Did the doctor/healer/chemist prescribe any medication?

    i) If yes, what medication/s: (list all, unsure=3, declined=99)

19. A) Since the bat festival, have you taken any medications?
    B) If yes, what medication/s: (list all, unsure=3, declined=99)

Follow-up Form
(Convalescent Blood Draw Visit)

20. Now I/we would like to ask you some questions about the symptoms you had when you were sick after the bat festival

**HISTORY OF ILLNESS**	**Have you had "*name specific symptom*"** (Yes = 1, No= 2, unsure= 3, declined =99)	**How many days ago did it start?** (if started today: code=00, NA=88, declined =99)	**How many days did the symptom last?** (if continuing until today, count current day as 1; NA= 88, declined=99)	**Did you have this symptom before or during the bat festival?** (Yes=1, No=2, unsure=3, NA=88, declined=99)
Fever:				
Chills:				
Nausea:				
Vomiting:				
Diarrhea:				
Abdominal pain:				
Cold:				
Conjunctivitis/red eye:				
Cough:				
Oral ulcers or cold sores:				
Sore throat:				
Difficulty breathing:				

Follow-up Form
(Convalescent Blood Draw Visit)

Chest pain:				
Muscle aches:				
Joint pain:				
Very tired/weak:				
Headache:				
Rash: Site___________________________				
Bleeding from gums or mouth				
**HISTORY OF ILLNESS**	**Have you had "*name specific symptom*"** (Yes = 1, No= 2, unsure= 3, declined =99)	**How many days ago did it start?** (if started today: code=00, NA=88, declined =99)	**How many days did the symptom last?** (if continuing until today, count current day as 1; NA= 88, declined=99)	**Did you have this symptom before or during the bat festival?** (Yes=1, No=2, unsure=3, NA=88, declined=99)
Blood spots in eyes (sclera) or on skin				
Stiff neck:				
Unbalanced/difficulty walking:				
Difficulty swallowing:				
Difficulty speaking:				
Difficulty hearing:				
Difficulty seeing:				

Follow-up Form
(Convalescent Blood Draw Visit)

Excessive fear/anxiety				
Agitated				
Tremors or convulsions:				
Altered or loss of consciousness:				
Paralysis:				
Other symptom 1, specify____________________				
Other symptom 2, specify____________________				

21. A) Since the last time we talked to you around 2 months ago, have you received rabies vaccination? Yes, No

    B) If yes, when were you given your last rabies vaccination?"

Those are all the questions I have for you. Thank you very much for your time and cooperation. We or personnel of the state Ministry of Health may need to contact you again if the survey is found to be incomplete. Results of this study will be reported to Ministry of Health representatives in your area.
