# Supplementary material for: Bat and Lyssavirus Exposure among Humans in Area that Celebrates Bat Festival, Nigeria, 2010 and 2013
Source: Emerg Infect Dis. 2020 Jul;26(7):1399–408. doi: 10.3201/eid2607.191016 (PMC7323560; doi:10.3201/eid2607.191016)
Supplement: Appendix 2 — Additional information about bat and lyssavirus exposure among humans in area that celebrates a bat festival, Nigeria, 2010 and 2013. [file 19-1016-Techapp-s2.pdf]

Article DOI: https://doi.org/10.3201/eid2607.191016

Bat and Lyssavirus Exposure among Humans in Area that Celebrates Bat Festival, Nigeria, 2010 and 2013

Appendix 2

Data Analysis

We summarized characteristics of enrolled households and persons represented among enrolled households in the 2010 and 2013 community surveys using descriptive statistics. The number and percentage of persons who had bat contact (overall and by type of contact) and who had eaten a bat were calculated among persons represented in the enrolled households from the community surveys. We analyzed demographics; household characteristics; bat-related activities; knowledge of rabies, bats, and animal bites; and history of rabies vaccination as potential associative factors in 3 different comparisons. Analyses included main household respondents who had ever had bat contact compared with those who didn't using logistic regression, a 2-sample *t*-test and Wilcoxon rank-sum test; participants in the 2010 community survey compared with those in the 2013 community survey who reported having ever had bat contact using generalized estimating equations (GEEs) with the logit link; and participants in the 2013 community survey and 2013 bat hunter survey who experienced febrile illness within 90 days of the bat festival versus those who did not, using GEEs with the logit link. The last 2 comparisons also looked at bat contact as a potential association. Odds ratios (ORs) with 95% confidence intervals were calculated for all 3 comparisons.

Main household respondents who participated in both the 2010 community survey and the 2013 community survey were excluded from the 2013 community survey results when analyses included both surveys. A response of "don't know" was considered "no" for the purpose of analysis. We analyzed data using SAS software (https://www.sas.com). A p value <0.05 was considered statistically significant.

Appendix 2 Table 1. Personal characteristics, household characteristics, practices, and knowledge of study participants who reported having ever had bat contact* in 2 community surveys and a bat hunter survey of bat exposures, Idanre area, Nigeria, 2010 and 2013.

Characteristic	2010 community survey, n (%)	2013 community survey, n (%)†	p value‡	OR (95% CI)‡	2013 bat hunter survey, n (%)
Ever had bat contact	72	131	-	-	21
Study participant type					
Main household respondent§	43 (60)	98 (75)	0.03	0.50 (0.27–0.92)	NA
Additional household respondent§	29 (40)	33 (25)	Ref	Ref	NA
Demographics					
Mean age (SD)	42 (16)	45 (18)	0.19	NC	51 (17)
Age range (y; min–max)	9–83	18–89	NC	NC	20–83
Median age (y; interquartile range)	40 (32–51.5)	43 (30-–60)	NC	NC	52 (38–64)
Age <25 y	8 (11)	18 (14)	0.61	0.78 (0.31–2.01)	2 (10)
Male	51 (71)	81 (62)	0.20	1.50 (0.81–2.79)	21 (100)
Education					
Some secondary or above	28 (39)	66 (50)	0.13	0.63 (0.34–1.14)	12 (57)
Completed secondary or above	18 (25)	45 (34)	0.18	0.64 (0.33–1.24)	8 (38)
Household characteristics					
Households	51	109			21
Persons in household					
<5 persons	12 (24)	28 (26)	0.77	0.89 (0.41–1.94)	3 (16)
<10 persons	34 (67)	72 (66)	0.94	1.03 (0.51–2.08)	10 (53)
Main material used to build house					
Adobe/mud	32 (63)	44 (40)	NP	NP	1 (5)
Cement/brick	18 (35)	65 (60)	NP	NP	20 (95)
Wood	1 (2)	0 (0)	NP	NP	0 (0)
Openings present in house that could allow bats to enter	31 (61)	58 (53)	0.37	1.36 (0.69–2.68)	1 (5)
Household with animals (pets or livestock)	34 (67)	60 (55)	0.17	1.63 (0.82–3.27)	14 (67)
Household with ≥1 animal (pet or livestock) that has been vaccinated against rabies	0 (0)	6 (10)	NP	NP	5 (36)
Types of bat contact					
Touch bat					
Ever touched	71 (99)	130 (99)	0.67	0.55 (0.03–8.96)	20 (95)
Last time touched ≤6 mo ago	29 (41)	62 (48)	0.38	0.76 (0.41–1.40)	16 (80)
Touch ≥2 times/y	39 (55)	8 (6)	<0.0001	18.43 (7.04–48.27)	1 (5)
Bite from bat					
Ever bitten	17 (24)	34 (26)	0.75	0.88 (0.41–1.89)	10 (48)
Last time bitten ≤6 mo ago	4 (24)	14 (41)	0.33	0.44 (0.08–2.32)	5 (50)
Bitten ≥2 times/y	11 (65)	0 (0)	NP	NP	1 (10)
Scratch from bat					
Ever scratched	23 (32)	41 (31)	0.89	1.05 (0.53–2.09)	15 (71)
Last time scratched ≤6 mo ago	6 (26)	18 (44)	0.24	0.45 (0.12–1.70)	8 (53)
Scratch ≥2 times/y	12 (52)	2 (5)	0.001	21.27 (3.83–118.07)	1 (7)
Other bat-related activities					
Participate in bat festival					
Ever participated	21 (44)	46 (35)	0.29	1.42 (0.74–2.72)	19 (90)
First time participated ≥20 y ago	0 (0)	30 (65)	NP	NP	10 (53)
Participate 2 times/yr	14 (70)	8 (18)	0.0002	10.79 (3.13–37.21)	1 (5)
Enter a bat cave or bat refuge					
Ever entered	30 (44)	41 (31)	0.07	1.73 (0.95–3.16)	18 (86)
Last time entered ≤6 mo ago	6 (20)	17 (41)	0.09	0.35 (0.11–1.17)	14 (78)
Enter ≥2 times/y	17 (57)	4 (10)	0.0002	12.10 (3.28–44.64)	0 (0)
Prepare a bat as food					
Ever prepared	64 (89)	108 (82)	0.24	1.70 (0.70–4.16)	18 (86)
Last time prepared ≤6 mo ago	31 (50)	64 (59)	0.25	0.69 (0.36–1.30)	14 (78)
Prepare ≥2 times/y	39 (61)	8 (7)	<0.0001	19.50 (7.78–48.85)	1 (6)
Eat a bat					
Ever eaten	66 (92)	113 (86)	0.29	1.75 (0.62–4.94)	21 (100)
Last time eaten <1 mo ago	5 (8)	63 (56)	<0.0001	0.07 (0.02–0.23)	16 (76)
Eat ≥2 times/y	43 (65)	12 (11)	<0.0001	15.74 (6.43–38.48)	2 (10)
Rabies knowledge					

Characteristic	2010 community survey, n (%)	2013 community survey, n (%)†	p value‡	OR (95% CI)‡	2013 bat hunter survey, n (%)
Indicated animal bites as mechanism of rabies transmission	44 (61)	76 (58)	0.70	1.14 (0.59–2.18)	19 (90)
Described rabies as severe	46 (65)	80 (61)	0.65	1.17 (0.58–2.35)	18 (86)
Identified bats as a rabies source	2 (3)	3 (2)	0.83	1.22 (0.20–7.47)	0 (0)
Identified dogs as a rabies source	51 (71)	78 (60)	0.13	1.65 (0.87–3.14)	19 (90)
If bitten or scratched by a bat					
Wash wound with soap and water	9 (13)	7 (5)	0.07	2.55 (0.92–7.07)	1 (5)
Seek medical care	13 (18)	45 (35)	0.01	0.42 (0.22–0.83)	1 (5)
Seek a traditional healer or pray	2 (3)	5 (4)	0.77	0.72 (0.08–6.50)	1 (5)
Do nothing	38 (54)	62 (48)	0.50	1.26 (0.64–2.48)	18 (86)
If bitten by a potentially rabid animal					
Wash wound with soap and water	4 (6)	1 (1)	0.07	7.65 (0.86–68.39)	1 (5)
Seek medical care	53 (74)	85 (65)	0.20	1.51 (0.80–2.85)	9 (43)
Seek a traditional healer or pray	3 (4)	6 (5)	0.90	0.91 (0.20–4.07)	0 (0)
Do nothing	3 (4)	29 (22)	0.002	0.15 (0.05–0.51)	10 (48)
History of rabies vaccination	1 (1)	2 (2)	0.94	0.91 (0.08–9.86)	1 (5)
Aware that bats can cause disease other than rabies	2 (3)	9 (7)	0.25	0.39 (0.08–1.93)	1 (5)
Know of reports of illness as a result of bats or being in bat cave	1 (1)	4 (3)	0.48	0.45 (0.05–4.09)	0 (0)

*Bat contact was defined as having touched a bat, having been bitten by a bat, or having been scratched by a bat.
†Ten of the 264 main household respondents participated in both the 2010 community survey and the 2013 community survey. They were deleted from the 2013 community survey data.
‡NA, not applicable; NC, not calculated; NP, logistic regression could not be performed due to zero cells.
§Main household respondents are adults or mature minors (persons aged 13–17 y who were married, had children, or provided for their own livelihood) present at the time of household visit who provided consent to participate in the survey; the main household respondent was the first person of the household to whom the study questionnaire was administered. Additional household respondents are other consenting or assenting household members who were immediately available to answer the study questionnaire and either had previously had bat contact or had previously eaten a bat.

Appendix 2 Table 2. Serologic testing of humans for lyssavirus antibodies in two community surveys and a bat hunter survey of bat exposures, Idanre area, Nigeria, 2010 and 2013.

Lyssavirus type (species)	Rabies virus (CVS-11)	Rabies virus (CVS-11)	Duvenhage virus (South Africa, 1970)	Lagos bat virus (lineage B, Nigeria, 1956)	Lagos bat virus (lineage B, Nigeria, 1956)	Lagos bat virus (lineage D, isolate KE576, Kenya, 2010)	Shimoni bat virus (Kenya, 2009)	Mokola virus (South Africa, 1998)	West Caucasian bat virus (Caucasus region, 2002)
Lyssavirus phylogroup	I	I	I	II	II	II	II	II	Undetermined
Sampling scheme	2013 community survey; 2013 bat hunter survey	2013 follow-up survey	2010 community survey	2010 community survey; 2013 community survey; 2013 bat hunter survey	2013 follow-up survey	2010 community survey	2010 community survey	2010 community survey	2010 community survey
Number of study participants tested	200	130	103	301	132	101	96	92	97
Number of study participants with detectable neutralizing antibodies	2	1	0	0	0	0	0	0	0

Appendix 2 Table 3. List of serologic testing results for lyssavirus antibodies among *Rousettus aegyptiacus* bats roosting in caves used in a bat festival, Idanre area, Nigeria, 2013.*

	Lyssavirus type (species)				
Bat ID	Duvenhage virus (South Africa, 1970)	Lagos bat virus (lineage B, Nigeria, 1956)	Shimoni bat virus (Kenya, 2009)	Mokola virus (South Africa, 1998)	Ikoma lyssavirus (Tanzania, 2009)
bat006	Neg	Neg	Neg	Pos	Neg
bat007	Neg	Neg	Neg	Neg	Neg
bat009	Neg	Neg	ND	Pos	Neg
bat011	Neg	Neg	Neg	Neg	Neg
bat012	Neg	Pos	Pos	Pos	Neg
bat015	Neg	ND	Pos	Pos	Neg
bat016	Neg	ND	Neg	Neg	Neg
bat019	Neg	ND	Pos	Pos	Neg
bat021	Neg	Pos	Pos	Pos	Neg
bat022	Neg	Neg	Neg	Neg	Neg
bat026	Neg	Pos	Pos	Pos	Neg
bat027	Neg	ND	Pos	Pos	Neg
bat028	Neg	Neg	ND	Neg	Neg
bat029	Neg	Pos	Pos	Pos	Neg
bat030	Neg	Pos	Pos	Neg	Neg
bat031	Neg	Neg	Neg	Neg	Neg
bat033	Neg	ND	ND	Neg	ND
bat035	Neg	ND	ND	ND	Neg
bat036	Neg	Pos	Pos	Pos	Neg
bat037	Neg	ND	ND	ND	ND
bat038	Neg	Neg	Neg	Neg	Neg
bat039	Neg	Neg	Pos	Pos	Neg
bat040	Neg	Neg	Neg	Neg	Neg
bat044	Neg	Pos	Pos	Pos	Neg
bat045	Neg	Neg	Neg	Neg	Neg
bat046	Neg	Pos	Pos	Pos	Neg
bat047	Neg	Pos	Pos	Pos	Neg
bat048	Neg	Pos	Pos	Pos	Pos
bat049	Neg	Pos	Neg	Pos	Neg
bat051	Neg	Pos	Neg	Pos	Neg
bat054	Neg	Pos	Pos	Pos	Neg
bat059	Neg	Neg	Neg	Neg	Neg
bat060	Neg	Pos	Neg	Neg	Neg
bat061	Neg	Pos	Pos	Pos	Neg
bat062	Neg	Neg	Neg	Neg	Neg
bat063	Neg	Pos	Neg	Pos	Neg
bat064	Neg	Neg	Neg	Neg	Neg
bat065	Neg	Neg	Neg	Neg	Neg
bat066	Neg	ND	Neg	Neg	Neg
bat067	Neg	Pos	Pos	Pos	Neg
bat068	Neg	Pos	Pos	Pos	Neg
bat070	Neg	Pos	Pos	Pos	Neg
bat071	Neg	Pos	Pos	Pos	Neg
bat072	Neg	Pos	Pos	Pos	Neg
bat073	Neg	ND	Pos	ND	Neg
bat074	Neg	Pos	ND	ND	Neg
bat075	Neg	Pos	Pos	Pos	Neg
bat076	Neg	Pos	Neg	Neg	Neg
bat077	Neg	Neg	Neg	Neg	Neg
bat078	Neg	Pos	Pos	Pos	Neg
bat079	Neg	ND	ND	ND	Neg
bat080	Neg	Pos	Pos	Pos	Neg
bat081	Neg	Neg	Neg	Pos	Neg
bat083	Neg	Neg	Neg	Neg	Neg
bat084	Neg	Pos	Neg	Pos	Neg
bat085	Neg	Neg	Neg	Neg	Neg
bat086	Neg	Pos	Pos	Pos	Neg
bat087	Neg	Pos	Pos	Pos	Neg
bat088	Neg	Neg	Neg	Pos	Neg
bat089	Neg	Neg	Neg	Pos	Neg
bat090	Neg	Neg	Neg	Neg	Neg
bat091	Neg	Neg	Neg	Neg	Neg
bat092	Neg	Pos	Pos	Pos	Neg

Bat ID	Lyssavirus type (species) Duvenhage virus (South Africa, 1970)	Lagos bat virus (lineage B, Nigeria, 1956)	Shimoni bat virus (Kenya, 2009)	Mokola virus (South Africa, 1998)	Ikoma lyssavirus (Tanzania, 2009)
bat097	Neg	Pos	Pos	Pos	ND
bat098	Neg	Neg	Neg	Neg	Neg
bat099	Neg	Neg	Neg	Neg	Neg
bat100	Neg	Pos	Pos	Pos	Neg

*A total of 211 bats were collected: 120 bats during September 2010 (112 *Rousettus aegyptiacus*, 8 *Hipposideros gigas*) and 91 during February 2013 (all *R. aegyptiacus*). This table displays only data on serologic testing for lyssaviruses among *R. aegyptiacus* bats in 2013; serum specimens were not available for all *R. aegyptiacus* bats. ND, not determined due to cytotoxicity or insufficient sample volume; Neg, negative for virus neutralizing antibodies (titer ≤1:10); Pos, positive for virus neutralizing antibodies (titer >1:10).
